# Targeting RNA G-quadruplex with repurposed drugs blocks SARS-CoV-2 entry

**DOI:** 10.1371/journal.ppat.1011131

**Published:** 2023-01-26

**Authors:** Qiyu Tong, Geng Liu, Xiongbo Sang, Xinyue Zhu, Xiaoli Fu, Chao Dou, Yue Jian, Jiani Zhang, Sailan Zou, Guixiang Zhang, Xiao Du, Dan Liu, Shiqian Qi, Wei Cheng, Yan Tian, Xianghui Fu

**Affiliations:** 1 Division of Endocrinology and Metabolism, National Clinical Research Center for Geriatrics, State Key Laboratory of Biotherapy and Cancer Center, West China Hospital, Sichuan University and Collaborative Innovation Center of Biotherapy, Chengdu, Sichuan, China; 2 Division of Pulmonary and Critical Care Medicine, State Key Laboratory of Biotherapy and Cancer Center, West China Hospital, Sichuan University, Chengdu, Sichuan, China; 3 Department of Gastrointestinal Surgery, West China Hospital, Sichuan University, Chengdu, Sichuan, China; 4 Division of Pulmonary and Critical Care Medicine, West China Hospital, Sichuan University, Chengdu, Sichuan, China; 5 Department of Urology, Institute of Urology (Laboratory of Reconstructive Urology), State Key Laboratory of Biotherapy and Cancer Center, West China Hospital, Sichuan University, Chengdu, Sichuan, China; Erasmus Medical Center, NETHERLANDS

## Abstract

The rapid emergence of SARS-CoV-2 variants of concern, the complexity of infection, and the functional redundancy of host factors, underscore an urgent need for broad-spectrum antivirals against the continuous COVID-19 pandemic, with drug repurposing as a viable therapeutic strategy. Here we report the potential of RNA G-quadruplex (RG4)-targeting therapeutic strategy for SARS-CoV-2 entry. Combining bioinformatics, biochemical and biophysical approaches, we characterize the existence of RG4s in several SARS-CoV-2 host factors. *In silico* screening followed by experimental validation identify Topotecan (TPT) and Berbamine (BBM), two clinical approved drugs, as RG4-stabilizing agents with repurposing potential for COVID-19. Both TPT and BBM can reduce the protein level of RG4-containing host factors, including ACE2, AXL, FURIN, and TMPRSS2. Intriguingly, TPT and BBM block SARS-CoV-2 pseudovirus entry into target cells *in vitro* and murine tissues *in vivo*. These findings emphasize the significance of RG4 in SARS-CoV-2 pathogenesis and provide a potential broad-spectrum antiviral strategy for COVID-19 prevention and treatment.

## Author summary

The rapid emergence of SARS-CoV-2 variants of concern, the complexity of infection, and the functional redundancy of host factors, underscore an urgent need for novel broad-spectrum antiviral strategies. RNA G-quadruplex (RG4), a non-canonical RNA secondary structure, has been recently implicated in the regulation of SARS-CoV-2 and host factors. Here, we further characterize the existence and functional importance of RG4s in several host factors, including ACE2. Moreover, we identify that Topotecan and Berbamine, two approved drugs, can function as RG4-stabilizing agents to repress RG4-containing host factors and protect cells and mice against SARS-CoV-2 pseudovirus entry. Together, this work highlights the significance of RG4 in SARS-CoV-2 pathogenesis and provide an attractive broad-spectrum antiviral strategy for COVID-19 prevention and control.

## Introduction

The unprecedented pandemic of coronavirus disease 2019 (COVID-19), caused by the novel severe acute respiratory syndrome coronavirus 2 (SARS-CoV-2), remains a global health crisis. Within the past two years, several SARS-CoV-2 variants of concern (VOCs) with escape mutations have emerged, which might evade the immunity induced by infection or vaccination, and thus jeopardize the effectiveness of currently approved vaccines and treatments [1], underscoring an urgent need for bioavailable broad-spectrum antivirals. Owing to reduced time-consuming and decreased developmental risk, drug repurposing has been substantially explored as a viable therapeutic strategy to rapidly harness the devastating COVID-19 threat, and several repurposed drugs have been approved for immediate clinical application [2]. However, some of the therapies had little or no beneficial effect on inpatients with COVID-19 in a large clinical trial [3]. To meet the above challenges from SARS-CoV-2 VOCs and limited effectivity of current medical interventions, a better understanding of molecular mechanisms underlying SARS-CoV-2 infection is urgently needed.

SARS-CoV-2 infection is initiated by binding of the spike (S) protein to its receptor, angiotensin-converting enzyme 2 (ACE2) at the target cell surface, followed by the fusion of viral and host membranes mediated by host proteases, such as TMPRSS2 [4], resulting in viral RNA release. In addition to ACE2 and TMPRSS2, an increasing number of host factors play an indispensable or redundant role in SARS-CoV-2 infection [5], including AXL [6], CD147 [7], CTSL [8], DPP4 [9], FURIN [10], and NRP1 [11,12]. In addition, several host factors may be relevant for SARS-CoV-2 infection, such as alpha7-nAChR [13], GOLGA7 [14], GRP78 [15], TLR4 [16], and ZDHHC5 [14]. Given the complexity of viral entry, the functional redundancy of host factors, and the multi-organ tropism of SARS-CoV-2, as well as the rapid emergence of VOCs, mechanism-based simultaneous targeting of multiple host factors, together with the virus *per se*, would offer a potentially more effective and broader paradigm for preventing and treating COVID-19.

Recent findings from ours and others revealed the presence of RNA G-quadruplex (RG4) in both SARS-CoV-2 and host factors (such as TMPRSS2), suggesting an attractive candidate for broad-spectrum antiviral therapy [17–20]. RG4 is a stable four-stranded conformation formed by self-recognition of guanines (Gs) to generate two or more layers of G-quartets. RG4 is enriched in viral RNA genomes [21] and also broadly exists in regions of biological relevance across human transcriptome [22]. RG4 is able to regulate gene expression at multiple levels, including RNA maturation, mRNA transport, localization, stability, and translation [23]. Correspondingly, RG4 is emerging as a promising target for human diseases [24], such as neuron degenerations [25], infectious diseases [26], cancers [27], and metabolic disorders [28]. Specifically, multiple RG4 structures have been reported in SARS-CoV-2 RNA genome by bioinformatics prediction and/or experimental identification, including the non-structural protein 3 (*Nsp3*) [20], *Nsp10* [17], *S* [18], and nucleocapsid (*N*) genes [19], indicating a potential role in viral replication and assembly. Furthermore, we recently showed that a RG4 structure within *Tmprss2* can significantly repress *Tmprss2* translation and prevent SARS-CoV-2 entry, exemplifying the regulation and importance of RG4 on host factors and virus infection [17]. More intriguingly, the RG4-stabilizing ligand pyridostatin (PDS) is capable of attenuating SARS-CoV-2 entry both *in vitro* and *in vivo*. These results collectively suggest that simultaneous targeting RG4 in both SARS-CoV-2 genome and host factors may provide a novel and powerful strategy to overcome the dilemma of COVID-19 pandemic. To explore this idea, the existence of RG4 in newly characterized host factors and the identification of RG4-stabilizing ligands from repurposed drugs merit careful investigation.

In this study, we determine the potential of RG4 for a series of SARS-CoV-2 host factors and characterize its existence in several molecules, including ACE2, AXL, and FURIN. Using *in silico* virtual docking method, we then identify topotecan (TPT) and berbamine (BBM), two clinical approved drugs, as RG4-stabilizing agents with repurposing potential for COVID-19. Both TPT and BBM can stabilize RG4 formation and reduce the protein level of RG4-containing host factors, including ACE2, AXL, FURIN, and TMPRSS2. More intriguingly, TPT and BBM efficiently block SARS-CoV-2 pseudovirus entry into cells and mouse tissues. These results not only highlight the significance of RG4 in SARS-CoV-2 pathogenesis, but also provide a new mechanism-based broad antiviral strategy for COVID-19 prophylactic and therapeutic.

## Results

### RG4 exists in multiple SARS-CoV-2 host factors

**Fig 1:**
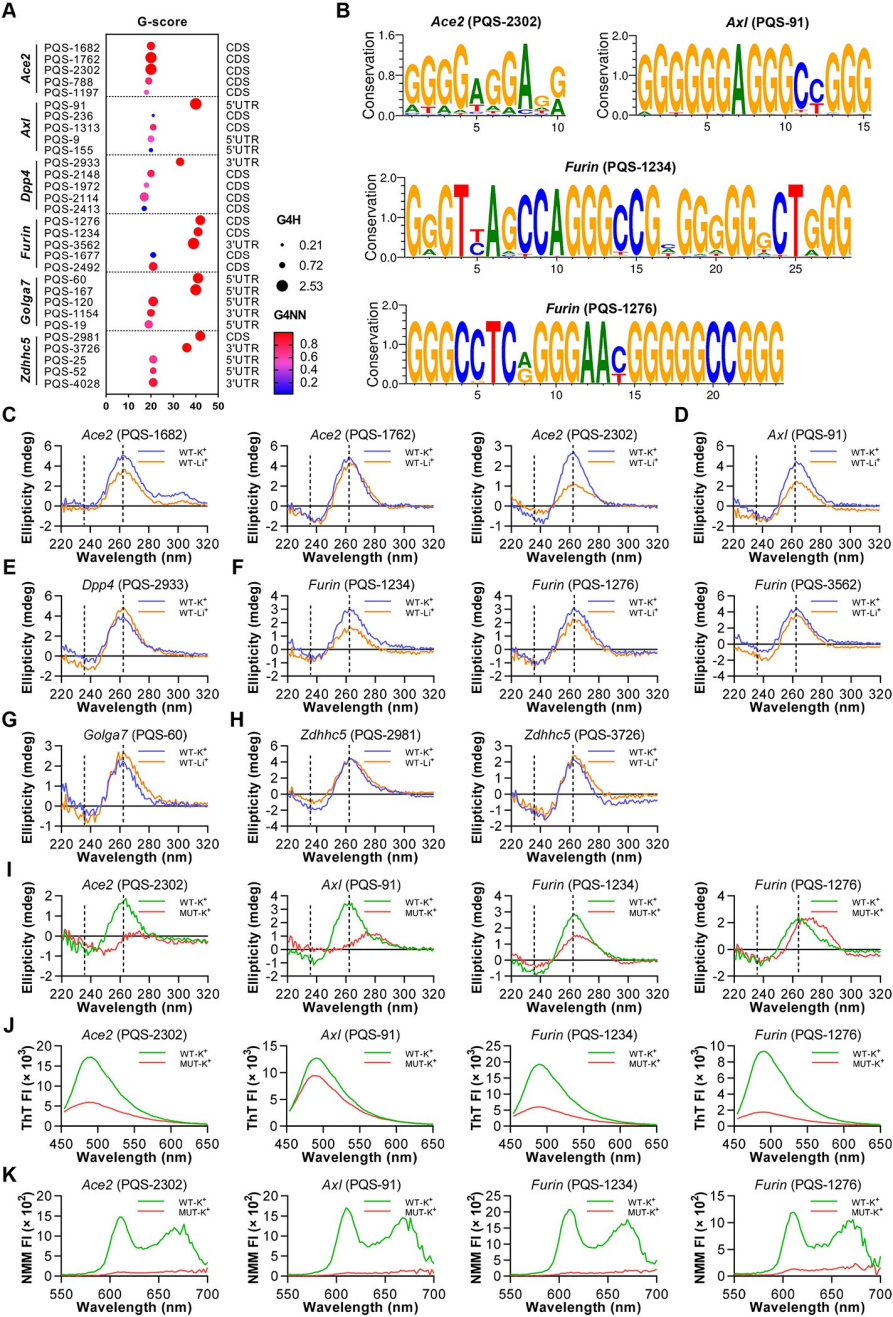
Characterization of RG4s in SARS-CoV-2 host factors. (A) RG4 potential of SARS-CoV-2 host factor mRNAs. Prediction results are shown with multi-parameter analysis of G-sore, G4H and G4NN. (B) Graphical representations of RG4 sequence conservation of PQSs in *Ace2*, *Axl* and *Furin*. The sequences were retrieved from the National Center for Biotechnology Information (NCBI) and aligned using WebLogo software. (C-H) CD spectrum for indicated PQSs of *Ace2* (C), *Axl* (D), *Dpp4* (E), *Furin* (F), *Golga7* (G), and *Zdhhc5* (H) under KCl or LiCl conditions. (I-K) CD spectrum (I), ThT (J) and NMM (K) fluorescence emission spectra for wild-type (WT) or mutated (MUT) of *Ace2* PQS-2302 (first panel), *Axl* PQS-91 (second panel), and *Furin* PQS-1234 (third panel) and PQS-1276 (fourth panel) under KCl conditions.

We previously reported a role of RG4 in TMPRSS2 expression and function [17]. Recently, numerous proviral host factors have been suggested to participate in SARS-CoV-2 entry [5,9,29]. Herein, we first reinforced the implication of RG4 in a series of newly identified SARS-CoV-2 host factors. To this end, we performed *in silico* analysis to define putative RG4 sequences (PQSs) in these host factors (S1A Fig and S1 Table). The combined multiparameter analysis of G-sore, G4H and G4NN revealed 11 PQSs with higher scores in 6 host factors, namely *Ace2* (PQS-1682, PQS-1762 and PQS-2302), *Axl* (PQS-91), *Dpp4* (PQS-2933), *Furin* (PQS-1234, PQS-1276 and PQS-3562), *Golga7* (PQS-60), and *Zdhhc5* (PQS-2981 and PQS-3726) (Fig 1A and S1 Table). These PQSs are also presented in various mammals, suggesting strong evolutionary conservation (Figs 1B and S1B).

These 11 PQSs were then used for experimental verification via circular dichroism (CD) spectroscopy, a standard method for characterizing G-quadruplex (G4) topology. K^+^, but not Li^+^, is required for the stabilization and maintenance of RG4 structure. 5 PQSs (PQS-1682, PQS-2302, PQS-91, PQS-1234 and PQS-1276) displayed a characteristic spectrum of a parallel G4 structure with ellipticity maximum and minimum at 264 and 238 nm, respectively, in the presence of KCl, while this pattern was diminished in LiCl buffer (Fig 1C–1H). In contrast, the remaining PQSs showed comparable CD spectrum in both KCl and LiCl buffer, indicative of reduced potential for RG4 formation (Fig 1C–1H). Two G4-binding fluorescent dyes, Thioflavin T (ThT) and N-methyl mesoporphyrin IX (NMM), were applied to further confirm the capability of these 5 PQSs for RG4 formation based on the fluorescence intensity. The fluorescence of both ThT and NMM for PQS-2302, PQS-91, PQS-1234 and PQS-1276 were higher in the presence of KCl than that of LiCl, albeit the difference of NMM fluorescence for PQS-91 was slight with unknown reason (S1C and S1D Fig). PQS-1682 displayed relatively weak RG4 features, as evidenced by ThT and NMM fluorescence probes (S1C and S1D Fig). Based on the combined results of three different analyses, 4 PQSs (PQS-2302 of *Ace2*, PQS-91 of *Axl*, and PQS-1234 and PQS-1276 of *Furin*) were chosen for further analysis.

Next, RG4-mutant RNAs were recruited to verify the sequence dependency of these PQSs for RG4 formation (S1E Fig). Under 150 mM KCl condition, G4-mutant (MUT) RNAs of these 4 PQSs had varying degrees of shift in CD peak wavelengths compared with wide type (WT) RNAs (Fig 1I). Importantly, both ThT and NMM assays recapitulated these observations (Fig 1J and 1K).

Together, these results not only suggest the presence of RG4 in multiple SARS-CoV-2 host factors, but also exemplify the potential of PQS-2302 in *Ace2*, PQS-91 in *Axl*, and PQS-1234 and PQS-1276 in *Furin*, to form RG4 structure *in vitro* (S1F Fig).

### A drug-repurposing screening identifies TPT and BBM as RG4-targeting agents

**Fig 2:**
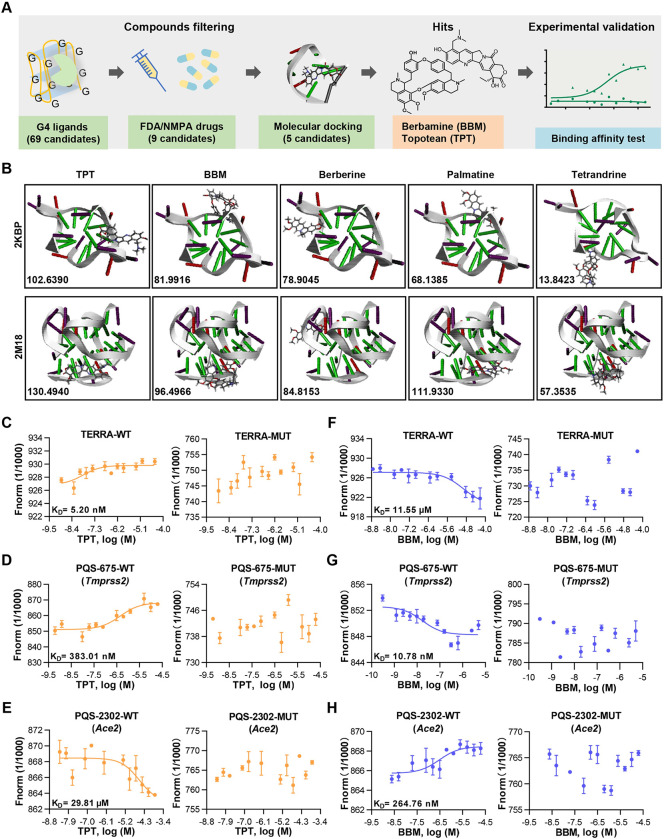
Filtering and verification of RG4-binding repurposed drugs. (A) Flow chart depicting the process of *in silico* identification and *in vitro* verification of RG4-based drugs. The number of drug candidates after each filtering step is shown, and TPT and BBM are finally identified. (B) Molecular docking of five drug candidates with TERRA-derived RG4 structures (PDB: 2KBP & 2M18) using Discovery Studio software. (C-H) Binding affinity of TPT (C-E) and BBM (F-H) to the WT (left panel) or mutant (right panel) RG4 RNA of TERRA (C, F), PQS-675 of *Tmprss2* (D, G), and PQS-2302 of *Ace2* (E, H) by microscale thermophoresis (MST) analysis. The dose−response curve for interactions was fitted with the dissociation constant (KD) model. Data are shown as mean ± SEM, n = 3. Fig 2A was created using Microsoft PowerPoint and Adobe Illustrator.

Our data presented in this and previous studies [17] collectively demonstrated the existence of RG4 in at least four host factors (*Ace2*, *Axl*, *Furin*, and *Tmprss2*) that are considered as potential therapeutic targets for COVID-19. Given the enrichment of RG4 in both SARS-CoV-2 genome and host factors, as well as the inhibitory effect of RG4 on gene expression, we hypothesized that RG4 stabilization might represent as an attractive broad-spectrum strategy to prevent and treat COVID-19. To explore this, we initially taken an unbiased approach to identify potential G4-binding compounds by consulting a G4 ligand library [30] and searching literature. This combined analysis identified a list of 69 pharmacological compounds with G4-binding potential, including 9 drugs approved by US Food and Drug Administration (FDA) and/or National Medical Products Administration (NMPA) (Fig 2A and S2 Table).

Given that drug repurposing has been extensively used to meet the unmet need for COVID-19, we focused on those 9 approved drugs. Among them, adriamycin, epirubicin, mitoxantrone, and bleomycin were excluded from further investigation, due to their severe side effects on heart and/or lung function [31–33]. For the remaining 5 drugs, we utilized an *in silico* virtual docking method to assess the interaction between the drug and RG4 structure. Because the high-order structures of RG4 sequences identified in this study remained unknown, two canonical RG4 structures formed by human TERRA sequences [34,35] were obtained from PDB (2KBP and 2M18) and used for docking stimulation. Among the drugs analyzed, TPT and BBM ranked as the top 2 candidates with great potential to bind both 2KBP and 2M18 (Fig 2A and S3 Table), and thus they were chosen for further study.

To validate the above *in silico* results, the TERRA, PQS-2302 of *Ace2*, and PQS-675 of *Tmprss2* were fluorescently labelled with CY5 and used to evaluate the RG4-binding ability of TPT and BBM via microscale thermophoresis (MST) (Fig 2C–2H). The sigmoid binding curves verified the high-affinity binding of these two drugs with both canonical and newly characterized RG4 structures. Intriguingly, the binding affinity of BBM for PQS-675 (*Tmprss2*) (dissociation constant (K_D_) = 10.78 nM) and PQS-2302 (*Ace2*) (K_D_ = 264.76 nM) was about 1000-fold and 40-fold higher than that of the TERRA (K_D_ = 11.55 μM), respectively (Fig 2F–2H). Importantly, these interactions were diminished by the mutation of RG4 sequences (Fig 2C–2H), supporting the direct binding of TPT and BBM to these RG4s.

Taken together, the combined computational and experimental analyses demonstrate that both TPT and BBM can directly bind to RG4s in the host factors and suggest a potential role in SARS-CoV-2 entry.

### TPT and BBM inhibit the expression of SARS-CoV-2 host factors

**Fig 3:**
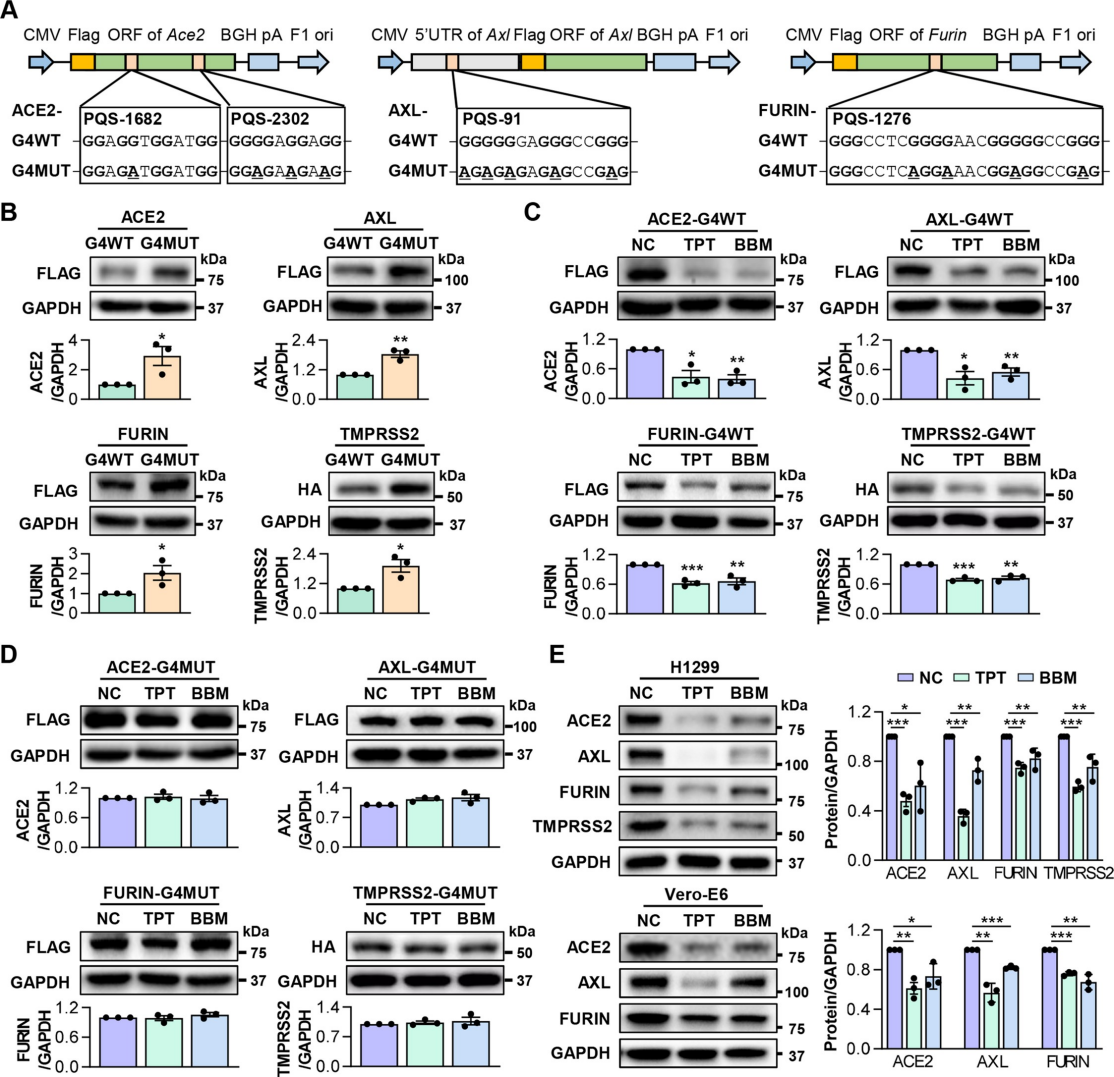
TPT and BBM repress RG4-containing host factors’ expression. (A) A schematic diagram of ACE2/AXL/FURIN-G4WT and their corresponding G4MUT plasmids. For ACE2, full-length ORF of *Ace2* (G4WT) or PQS-1682/PQS-2302 G4-mutant *Ace2* (G4MUT) were inserted into the MCS of pcDNA3.1 vector. For AXL, full-length 5’UTR of *Axl* (G4WT) or PQS-91 G4-mutant *Axl* (G4MUT) were inserted prior to the *Axl* ORF. For FURIN, full-length ORF of *Furin* (G4WT) or PQS-1276 G4-mutant *Furin* (G4MUT) were inserted into the multiple cloning site (MCS) of pcDNA3.1 vector. (B) Protein levels of FLAG-AXL, FLAG-ACE2, FLAG-FURIN and HA-TMPRSS2 in H1299 cells transfected with their respective G4WT or G4MUT plasmids. (C, D) Protein levels of FLAG-AXL, FLAG-ACE2, FLAG-FURIN and HA-TMPRSS2 in H1299 cells transfected with their respective G4WT (C) or G4MUT (D) plasmids in the presence of TPT or BBM. (E) Protein levels of endogenous AXL, ACE2, FURIN and TMPRSS2 in H1299 (top panel) and Vero-E6 (bottom panel) cells treated with TPT or BBM. ImageJ quantification of the target/GAPDH ratio is shown. Data are shown as mean ± SEM, n = 3. *p < 0.05, **p < 0.01, ***p < 0.001 (Two-tailed Student’s t test).

Next, we determined whether TPT and BBM could repress the expression of SARS-CoV-2 host factors via stabilizing RG4 structures. Because the aforementioned PQS-1682, PQS-2302 and PQS-1276 are located in the open reading frame (ORF), we generated pcDNA3.1 plasmids containing the full-length ORF of *Ace2* and *Furin*, respectively. In parallel, their RG4 mutants were generated, in which guanines in each G-tracts of PQS-1682, PQS-2302 and PQS-1276 were substituted with adenines (Fig 3A), which were designed to eliminate the RG4 formation with synonymous substitution. Since PQS-91 is localized in the 5’UTR of *Axl*, we inserted the full-length or RG4 region mutant 5’UTR of *Axl* before its ORF (Fig 3A). These plasmids were transfected into H1299 cells respectively, and cells were harvested 24 hrs later for QPT-PCR and western blot analysis. Transfection of G4WT and G4MUT plasmids led to comparable increases in the mRNA levels of *Ace2*, *Axl*, *Furin* and *Tmprss2* (S2A Fig). However, protein levels of ACE2, AXL, and FURIN, as well as TMPRSS2 described in our previous study [17], were higher in G4MUT cells than in G4WT cells (Figs 3B and S2B), suggesting a post-transcriptionally inhibitory effect of RG4 structure on host factor expression. Notably, TPT and BBM led to considerable decreases in the protein levels of ACE2, AXL, FURIN, and TMPRSS2 in G4WT cells (Figs 3C and S2B). This inhibition was abolished in G4MUT cells (Figs 3D and S2B), suggesting that TPT/BBM-mediated suppression on host factor expression depends on RG4 formation.

Moreover, we confirmed the effect of TPT and BBM on the endogenous expression of host factors in two SARS-CoV-2 susceptible cell lines, namely H1299, and Vero-E6 (lack of endogenous *Tmprss2*). Western blot analysis showed that both TPT and BBM markedly reduced the protein levels of these RG4-containing host factors (Figs 3E and S2B). In contrast, TPT and BBM appeared to have no effect on the mRNA levels of these factors, as exemplified by *Tmprss2* (S2C and S2D Fig), consistent with the post-transcriptional regulatory mode of RG4 [24], as well as our previously identified RG4-mediated translational inhibition on *Tmprss2* [17].

Altogether, these results suggest that TPT and BBM can potently inhibit the expression of several SARS-CoV-2 host factors at the post-transcriptional level through stabilizing RG4 formation.

### TPT and BBM block both SARS-CoV-2 and VOCs pseudovirus entry into target cells

**Fig 4:**
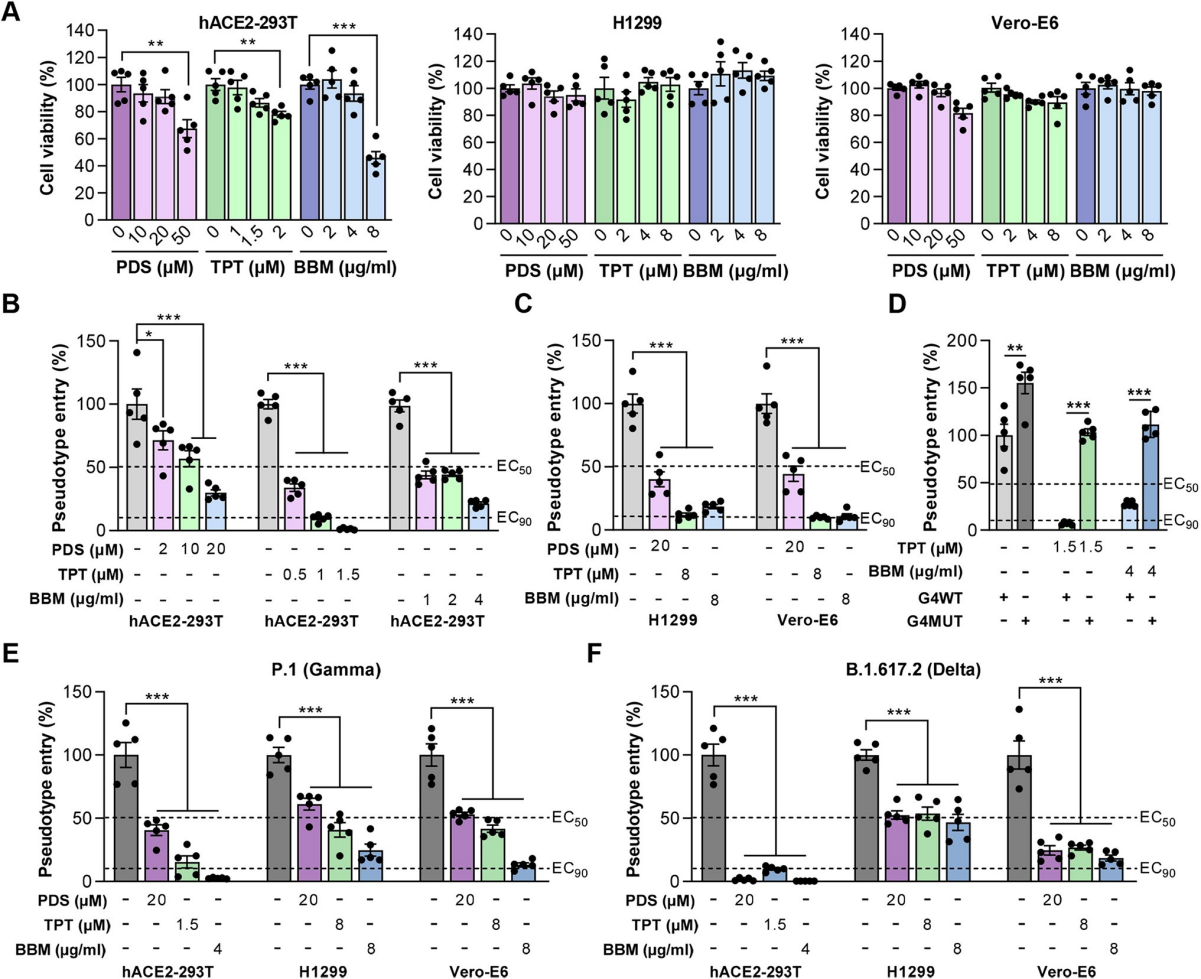
TPT and BBM block SARS-CoV-2 pseudovirus entry *in vitro*. (A) Relative cell viability of hACE2-293T (left panel), H1299 (middle panel), and Vero-E6 (right panel) cells treated with the indicated concentrations of PDS, TPT or BBM. (B, C) SARS-CoV-2 pseudovirus entry efficiency in hACE2-293T (B), H1299 (C) and Vero-E6 (C) cells treated with the indicated concentrations of PDS, TPT, or BBM. Cells were pre-treated with indicated compounds and then infected with VSV-SARS-2-S-luc. 48 hrs post infection, pseudovirus entry was assessed by luciferase activity (normalization against untreated cells). (D) SARS-CoV-2 pseudovirus entry efficiency in hACE2-293T cells transfected with the plasmid pool of ACE2-/AXL-/FURIN-/TMPRSS2-G4WT or their corresponding G4MUT plasmids and stimulated with TPT or BBM. (E, F) Entry efficiency of P.1 (E) and B.1.617.2 (F) variants in hACE2-293T, H1299, and Vero-E6 cells treated with the indicated concentrations of PDS, TPT, or BBM. Data are shown as mean ± SEM, n = 5. *p < 0.05, **p < 0.01, ***p < 0.001 (Two-tailed Student’s t test).

Given the positive correlation between host factor expression and SARS-CoV-2 entry, we subsequently assessed the influence of TPT and BBM in preventing SARS-CoV-2 pseudovirus entry. hACE2-293T cells were pretreated with an increasing dose of these two repurposed drugs (TPT and BBM), as well as PDS that was used as a positive control stabilizing RG4 and blocking virus entry [17], for 5 hrs prior to infection with a recently established SARS-CoV-2 pseudovirus system [36]. Under the precondition of safety concentration (Fig 4A), both TPT and BBM significantly reduced pseudovirus entry efficiency in a dose-dependent manner (Fig 4B). Intriguingly, TPT and BBM with low dosage exhibited higher inhibition efficiency than the positive control PDS. Specifically, TPT (1.5 μM) dramatically blocked pseudovirus entry into hACE2-293T cells, leading to over 20-fold greater efficiency than that of PDS (20 μM). These observations were recapitulated in H1299 and Vero-E6 cells (Fig 4C). Compared with PDS, TPT and BBM resulted in about a 2-fold and 4-fold decrease in pseudovirus entry efficiency in H1299 and Vero-E6 cells, respectively, indicating a cellular heterogeneity of virus entry inhibition. These results collectively suggest a broad-spectrum antivirus capability of BBM and TPT across different cells.

Next, we tested whether the inhibitory impact of TPT and BBM on SARS-CoV-2 pseudovirus entry relies on RG4s in host factors. In line with enhanced protein levels (Fig 3B), simultaneously transfection of ACE2, AXL, FURIN and TMPRSS2-G4MUT plasmids into hACE2-293T cells led to an increase in pseudovirus entry efficiency, compared with their corresponding G4WT plasmids (Fig 4D). Moreover, both TPT and BBM markedly inhibited pseudovirus entry in G4WT cells, and this inhibition was significantly, but not completely, abolished in G4MUT cells (Fig 4D), suggesting that RG4 may predominantly mediate the effects of these two chemicals.

The emergence of SARS-CoV-2 VOCs has raised concern that some of these new strains may evade current vaccines. Unlike viral genes, host genes often have a low propensity to mutate, representing an attractive therapeutic strategy for combating SARS-CoV-2 VOCs. P.1 (Gamma) and B.1.617.2 (Delta) are two dominant circulating variants that led to worldwide concern in 2021 [1]. Interestingly, TPT and BBM significantly reduced both P.1 (Gamma) and B.1.617.2 (Delta) pseudovirus entry efficiency in all three SARS-CoV-2 susceptible cells, albeit considerable variations were observed in different cell types (Fig 4E and 4F).

Altogether, these data underline the potential of RG4-based drugs, especially TPT and BBM, as a pan-variant antiviral strategy against emerging SARS-CoV-2 VOCs.

### TPT and BBM protect mice against SARS-CoV-2 pseudovirus entry

**Fig 5:**
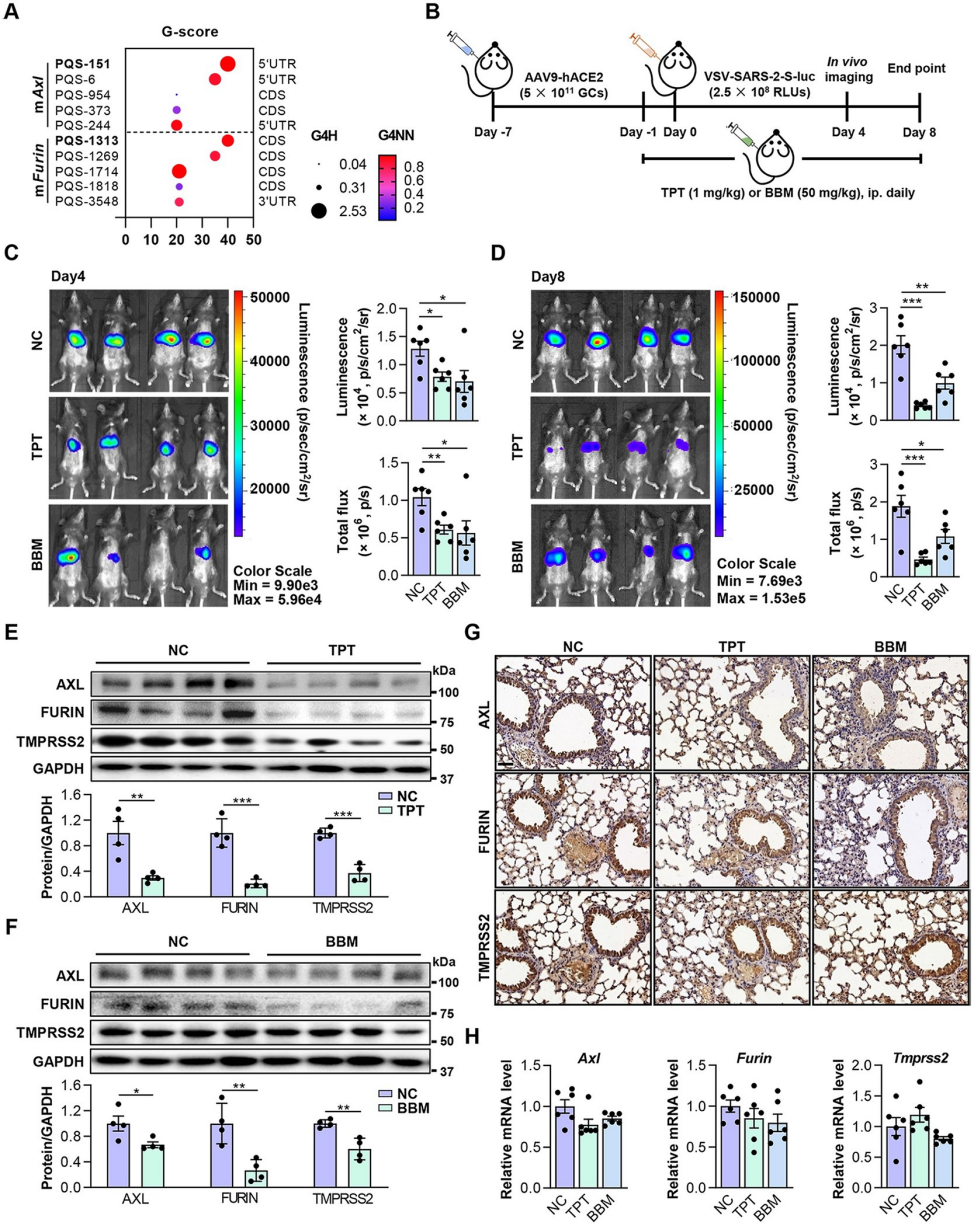
TPT and BBM impede SARS-CoV-2 pseudovirus entry in mice. (A) RG4 potential of mouse *Axl* and *Furin* mRNAs. PQSs conserved between human and mouse are indicated in **bold**. (B) Schematic of a mouse model for SARS-CoV-2 pseudovirus infection. C57BL/6J mice were transduced intrathoracic with the human ACE2-expressing adenovirus (AAV9-hACE2) and infected with VSV-SARS-2-S-luc after 7 days. Mice received daily peritoneal injections of TPT (1 mg/kg), BBM (50 mg/kg), or vehicle for 9 days from -1 day before VSV-SARS-2-S-luc infection. Following measurements were performed on day 4 and 8 post-infection (n = 6). (C, D) Representative photos of mice on day 4 (C) and 8 (D) post-infection (n = 6). Relative levels of bioluminescence are shown in pseudocolours, with blue and red representing the weakest and strongest photon fluxes, respectively. Photon emission from each mouse was quantified as luminescence (p/s/cm2/sr) and total flux (p/s) post imaging, respectively. (E, F) Protein levels of AXL, FURIN and TMPRSS2 in lungs of VSV-SARS-2-S-luc-infected mice treated with TPT (E) or BBM (F) (upper panel) (n = 4). ImageJ quantification of the target/GAPDH ratio is shown (bottom panel). (G) IHC staining for AXL, FURIN and TMPRSS2 in paraffin lung sections. Scale bars: 50 μm. (H) mRNA levels of *Axl, Furin*, and *Tmprss2* in lungs of VSV-SARS-2-S-luc-infected mice treated with TPT or BBM (n = 6). Data are shown as mean ± SEM, n = 4–6. *p < 0.05, **p < 0.01, ***p < 0.001 (Two-tailed Student’s t test). Fig 5B was created using Microsoft PowerPoint and Adobe Illustrator.

Finally, we investigated the effect of TPT and BBM on SARS-CoV-2 pseudovirus entry in mouse models. As mentioned above, PQSs in human *Axl* and *Furin* are highly conserved in the mouse genome (Fig 5A and S4 Table). Notably, the sequence of PQS-91 in human *Axl* is identical to that of PQS-151 in mouse homolog (S3A Fig). Additionally, we have previously shown that the murine *Tmprss2* is also regulated by RG4 [17]. The conserved existence and function of RG4 within SARS-CoV-2 host factors (*Axl*, *Furin*, and *Tmprss2*) makes it possible to determine the impact of RG4-targeting drugs on SARS-CoV-2 pseudovirus entry *in vivo*. It is worth noting that the mouse ACE2 is incapable of mediating SARS-CoV-2 entry [37], therefore the potential contribution of *Ace2* RG4 on SARS-CoV-2 pseudovirus entry in mouse models was neglected in this study.

We then evaluated the effect of RG4-targeting agents (TPT and BBM) by using a recently established mouse model [17]. Briefly, C57BL/6J mice were injected with adeno-associated virus 9 (AAV9)-hACE2 and infected with VSV-SARS-2-S-luc 7 days after. From -1 day post infection (DPI), mice were administrated with TPT (1 mg/kg) or BBM (50 mg/kg) daily for 10 days (Fig 5B). *In vivo* fluorescent imaging was conducted at 4 and 8 DPI, respectively. Administration of TPT and BBM led to a 40% reduction in fluorescence signal at 4 DPI compared with the placebo (Fig 5C). At 8 DPI, there were 80% and 50% reduction for TPT and BBM treatment, respectively (Fig 5D), indicating decreased pseudovirus entry. In line with this, TPT- and BBM-treated mice had reduced AXL, FURIN and TMPRSS2 protein levels in mouse lungs, with TPT being more efficient on host factor inhibition (Fig 5E–5G). In parallel, TPT and BBM administration had no obvious effect on the mRNA levels of *Axl*, *Furin* and *Tmprss2* (Fig 5H), further supporting a post-transcriptional regulation on host factor expression. Consistent with previous reports [38,39], TPT- and BBM-treated mice did not have clinically significant adverse changes in histological or hematological parameters (S3B and S3C Fig), suggesting that these treatments were safe, and the above observations may not result from drug-induced toxicity.

Together, these results suggest that TPT and BBM may prevent SARS-CoV-2 pseudovirus entry *in vivo*, providing potential RG4-based therapeutic agents to beat COVID-19 pandemic.

## Discussion

**Fig 6:**
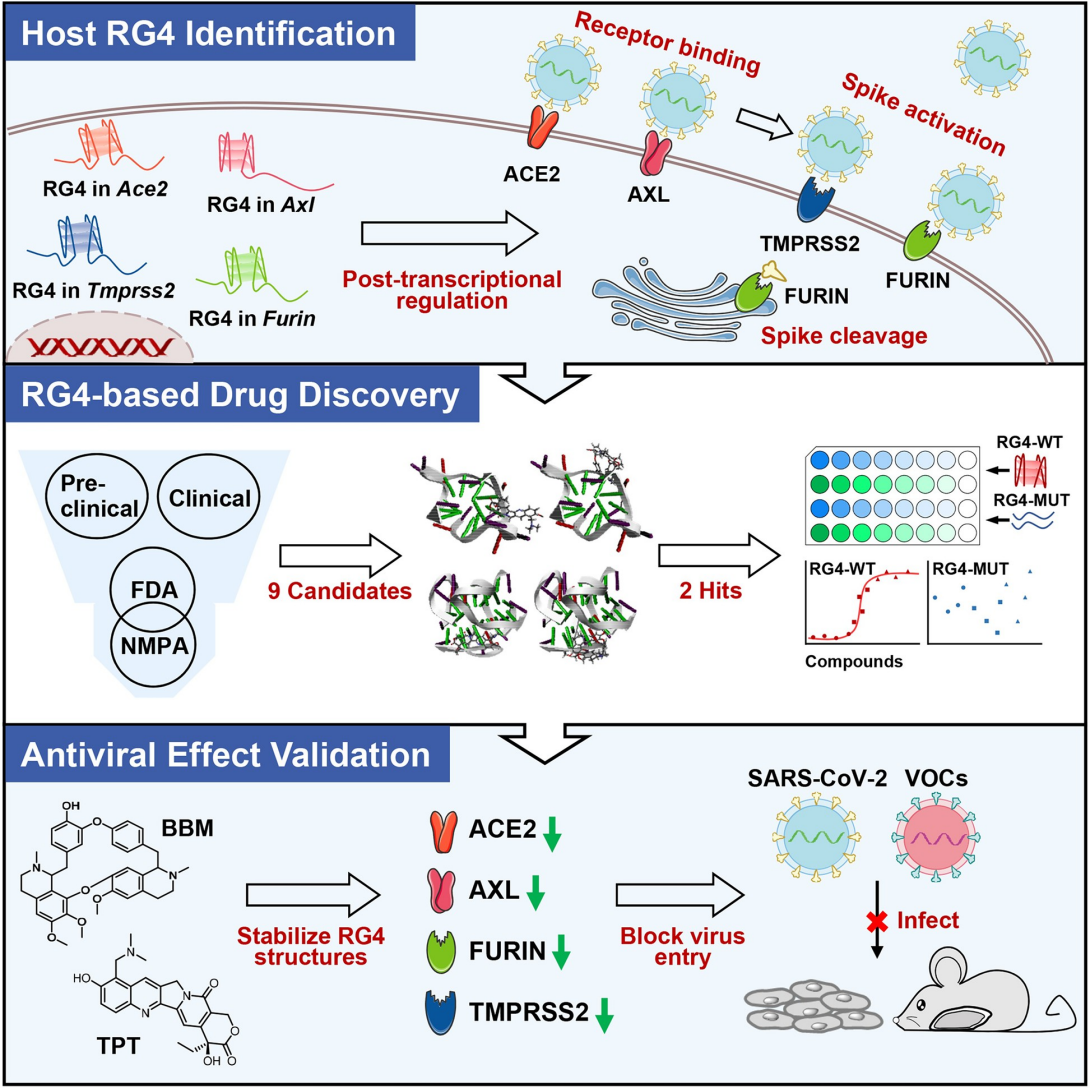
A schematic model of this article. Topotecan and Berbamine may block SARS-CoV-2 infection by suppressing RG4-containing host factors. Fig 6 was created using Microsoft PowerPoint and Adobe Illustrator.

In this study, we explore the potential of RG4-targeting therapeutics on SARS-CoV-2 infection. Several SARS-CoV-2 entry factors harbor multiple evolutionarily conserved PQSs with high potential for RG4 formation. Moreover, we identify that TPT and BBM, two approved drugs, can function as RG4-stabilizing agents to reduce the expression of these host factors. Intriguingly, both TPT and BBM are sufficient to prevent SARS-CoV-2 pseudovirus entry *in vitro* and *in vivo* (Fig 6). These findings emphasize the importance of RG4 in SARS-CoV-2 pathogenesis and highlight a fascinating strategy for COVID-19 prevention and treatment.

Current treatments for COVID-19 can roughly be divided into two categories: direct-acting antivirals and host-directed therapies [5,40]. Direct-acting antivirals, such as vaccines and antibodies, target the virus components and their efficacy are assumed to be weakened by viral mutations conferring resistance. Indeed, the emergence of SARS-CoV-2 VOCs represents as a major challenge for direct-acting antivirals. For example, the P.1 (gamma) and B.1.135 (beta) VOCs with S protein mutations markedly impaired the neutralizing capacity of the antibody cocktail (bamlanivimab and etesevimab) [41], while the currently dominating B.1.1.529 (omicron) VOC may reduce the efficacy of most monoclonal antibody treatments [42]. Conversely, host-directed therapies that target components of the host cell are not susceptible to virus mutations, making them more advantageous than direct-acting antivirals for harnessing VOCs. Currently, available host-directed therapies are quite limited and restricted to dampen infection-triggered deregulated inflammatory response [40]. Given the emergence of VOCs, as well as the complexity of viral entry and diversity of host factor usage, it is an unmet need to explore novel host-directed therapeutic strategies to beat the COVID-19 pandemic. Recently, we and others have suggested a regulatory role of RG4 in SARS-CoV-2 genome and *Tmprss2* [17–19, 43]. Here, we expanded the presence of RG4 in other newly identified host factors, including *Ace2*, *Axl*, and *Furin*. ACE2, a coronavirus receptor, is essential for the entry of SARS-CoV-2 into the host cell [4]. Interestingly, it has recently been indicated that AXL can facilitate SARS-CoV-2 entry into ACE2-deficient cells [6]. The cleavage of S protein by host proteases, such as FURIN [10,44], is a prerequisite for virus membrane fusion. In this regard, it is reasonable to imagine that RG4 may modulate many steps of SARS-CoV-2 infection. Of note, although *Ace2* PQS-2302 with moderate G-score is likely to form a non-canonical RG4 structure with two quarters (S1F Fig), its formation can significantly reduce the levels of ACE2 protein (Fig 3B–3E). Intriguingly, numerous PQSs with similar G-score were predicted in multiple host factors, including *Cd147*, *Ctsl*, *Nrp1*, *Alpha7-nAChR*, and *Tlr4* (S1A Fig), albeit they have not been experimentally investigated in this study. It is possible that some of them might have an ability to form RG4 structure and regulate SARS-CoV-2 infection. Taken together, the prevalent existence of RG4 in host factors, as well as in SARS-CoV-2 genome, strongly suggests a broad-spectrum antiviral strategy.

We conducted a series of experiments to explore the clinical implication of this attractive antiviral strategy. Repurposing of known drugs could substantially accelerate the deployment of new therapies for COVID-19, due to the validated side effects, and previously optimization for safety [45]. In this regard, we initially identified TPT and BBM, two approved drugs, as direct stabilizers of RG4 structure. A combination of *in silico* molecular docking and MST analysis demonstrated a direct interaction between these two drugs and representative RG4s within SARS-CoV-2 host factors. Importantly, TPT and BBM can potently reduce the protein levels of ACE2, AXL, FURIN, and TMPRSS2, likely due to their effect on stabilizing RG4 structure. Moreover, both TPT and BBM significantly prevented SARS-CoV-2 pseudovirus entry into all susceptible cell lines that we examined. Intriguingly, TPT- and BBM-mediated inhibition efficiency was much higher than PDS (Fig 4B and 4C), the latter had a comparable impact on SARS-CoV-2 pseudovirus entry with camostat, a known anti-COVID-19 drug, as we described previously [17]. Accordingly, administration of TPT and BBM in mice led to a reduction in pseudovirus entry *in vivo*, accompanied with obvious decreases in the protein levels of host factors containing RG4 PQSs, such as AXL, FURIN, TMPRSS2. Interestingly, comparative effects of BBM and TPT *in vitro* and *in vivo* appeared to be different. That is, BBM was more effective for inhibiting pseudovirus entry in cultured cells (Fig 4), but was less in mouse models than TPT (Fig 5). This observation might be associated with the differences of these two drugs in optimized dose, and the rate of drug absorption and metabolism *in vivo*, which awaits further investigation. Taken together, these results strongly suggest a potent inhibitory role of TPT and BBM in SARS-CoV-2 pseudovirus entry.

Several SARS-CoV-2 VOCs have been emerged since September 2020, which dominate the continuous pandemic. Gamma and Delta in 2021, and Omicron in 2022, have become the dominant circulating variant which reduced vaccine efficacy and worse infection outcomes [46]. Host-directed therapies provide a reduced chance of resistance development and the potential for broad action against viral variants [47]. Indeed, our results showed that BBM and TPT significantly reduced the entry efficiency of Gamma and Delta pseudovirus (Fig 4E and 4F), two available commercial variants when we executed the study. Of note, RG4 PQSs are also presented in the ORFs of several SARS-CoV-2 genes, including the *Nsp3* [20], *Nsp10* [17], *S* [18], and *N* genes [19]. Moreover, base conservation analysis showed that these PQSs remain unchanged in all dominating SARS-CoV-2 variants that we examined, including Alpha (B.1.1.7), Beta (B.1.351), Gamma (P.1), Delta (B.1.617.2), and Omicron (B.1.1.529, XBB, BQ.1, BA.5 and BA.2.75.2) (S4 Fig), suggesting that RG4-based therapeutics may also act as direct-acting antivirals. Additionally, it is well known that RG4 is enriched in numerous viruses [21,26], including Ebola virus [48], hepatitis C virus [49], human immunodeficiency virus [50], and SARS-CoV [51], and is extensively implicated in virus pathogenesis and infectious diseases. Given the broad existence of RG4 in both virus genome and host factors, as well as the low evolvement of RG4 sequences, RG4-targeting drugs, such as TPT and BBM, may act as broad-spectrum strategies to combat multiple virus diseases, even future viral pandemics.

The natural compound BBM is a critical anti-tumor drug that targets CAMKIIγ [38]. TPT, a FDA-approved topoisomerase 1 (TOP1) inhibitor, has been used in the treatment of small cell lung cancer [52]. Besides these traditional targets and effects, recent reports have suggested their regulatory roles in DNA G-quadruplex (DG4) formation. BBM has highly binding affinity toward the (GGA)_8_ [53] and telomere DG4s [54], while TPT can induce DG4 formation in the promoter of c-myb, an oncogene for many cancers [55]. In this study, we found that BBM and TPT are also capable of stabilizing RG4 structure, probably due to a similar folding topology shared by RG4 and DG4. However, elimination of RG4 structures in *Ace2*, *Axl*, *Furin* and *Tmprss2* was unable to completely abolish the inhibitory effect of TPT and BBM on SARS-CoV-2 pseudovirus entry (Fig 4D), suggesting a potential involvement of RG4-independent mechanisms. Indeed, during the preparation of the manuscript, both BBM and TPT have been implicated in the treatment of COVID-19. BBM can inhibit SARS-CoV-2 entry by compromising the transient receptor potential mucolipin channels (TRPMLs)-mediated endolysosomal trafficking of ACE2 [56], and blocking S protein-mediated membrane fusion [57]. In addition, BBM has been recently shown to repress SARS-CoV-2 prototypic and Delta variant infection, [58], consistent with our results. TPT may suppress SARS-CoV-2 infection-induced inflammation and death in mouse models [39], but its role in virus entry remains unclear. Moreover, there is an ongoing clinical trial to investigate the antiviral effect of TPT on COVID-19. Our results, together with these recent advances, strongly implicate the great potential of BBM and TPT in the prevention and treatment of COVID-19, although their underlying mechanisms, as well as potential side effects, merit further study.

Nevertheless, several limitations remain to be addressed in the future. For instance, the screening library of DG4/RG4 ligands used in this study is relatively small. With the recent development of a large database containing >3200 G4 ligands [59], it is highly anticipated that more potential RG4-stabilizing agents with antiviral activity would be characterized in the future. In addition, because the pseudovirus system can only mimic the processing of viral entry, it is of great interest to confirm the beneficial effect of BBM and TPT on the infection of authentic SARS-CoV-2 and its variants, including Omicron, in animal models and clinical trials.

In summary, this study identifies targeting RG4 as a broad-spectrum host-directed and direct-acting antiviral therapy and highlights the potential clinical translation of RG4-based drugs for COVID-19 prevention and treatment.

## Materials and methods

### Ethics statement

All animal related studies were approved by the Institutional Animal Care and Use Committee of West China Hospital, Sichuan University (protocol number: 20220125002).

### Animals

The C57BL/6J mice (male, 6- to 8- weeks) were purchased from Nanjing Biomedical Research Institute. Mice were maintained in grouped cages (22°C, 12 hrs light-dark cycle), and fed with standard chow diet and water ad libitum. All mouse related studies were approved by the protocols approved by the Institutional Animal Care and Use Committee of West China Hospital, Sichuan University.

### Cell culture

H1299 (CRL-5803) and Vero-E6 (CRL-1586) were obtained from the American Type Culture Collection (ATCC). hACE2-293T were constructed as previously described [17]. Cells were maintained in Dulbecco’s Modified Eagle Medium (Gibco, 10569044) supplemented with 10% fetal bovine serum (Gibco, 30044184) and 1% Penicillin-Streptomycin (Gibco, 15140122) at 37°C and 5% CO_2_.

### Oligonucleotides and antibodies

DNA and RNA oligonucleotides (S5 and S6 Tables) were purchased from Beijing TSINGKE Biological Technology and Shanghai GenePharma Corporation respectively. Antibodies used in this study were listed as follows: anti-ACE2 (Abcam, ab108252), anti-AXL (ABclone, A17874), anti-FLAG (ABclone, AE004), anti-FURIN (Abcam, ab183495), anti-GAPDH (Proteintech, 60004–1), anti-HA (CST, 3724S) and anti-TMPRSS2 (Abcam, ab280567).

### SARS-CoV-2 pseudovirus entry for mouse

Mouse ACE2 is incapable of mediating SARS-CoV-2 entry, thus mice were heterogeneously expressed of hACE2 via adenoassociated virus (hACE2-AAV9) (Delivectory Biosciences) and challenged with SARS-CoV-2 pseudovirus (Delivectory Biosciences). The SARS-CoV-2 pseudovirus VSV-SARS-2-S-luc consist of a lentiviral core with Renilla, and the SARS-CoV-2 S protein on its envelope. Therefore, cells/animals infected with pseudoviruses can be examined via luciferase activity. The SARS-CoV-2 pseudovirus mouse model was performed according to our previous study [17]. In brief, mice were injected with 5 × 10^11^ genomic copies (GCs) of AAV9-hACE2, and then challenged with 2.5 × 10^8^ relative light units (RLUs) of SARS-CoV-2-S-luc pseudovirus (Delivectory Biosciences) 7 days later. For drug intervention, from −1 to 8 DPI, mice were injected with TPT (1 mg/kg body weight, Cayman Chemical, 2076-91-7) and BBM (6 mg/kg body weight, MCE, HY-N0714), respectively, through intraperitoneal administration once a day.

### In vivo imaging

*In vivo* imaging measurements were performed on day 4 and day 8 post infection. During the measurement, mice were anesthetized using 30% isoflurane inhalation before imaging, and intraperitoneally injected with D-Luciferin (Promega, P1041) in PBS at a 150 mg/kg body weight dose. 5 to 10 min later, the bioluminescence imaging was captured using the IVIS Spectrum imaging system (PerkinElmer). Bioluminescence values are indicated as Total Flux and Luminescence via the Living Image Analyze 12.0 (PerkinElmer).

### Hematoxylin and eosin (H&E) staining

The fresh tissues of lung, liver, kidney, and heart from mice were fixed in 10% neutral-buffered formalin, embedded in paraffin and sectioned into 4 μm-thick. Then, these slides were stained with H&E for morphological analyses as described previously [60].

### Serum biochemistry

Blood samples were obtained by cardiac puncture and centrifuged at 3,000 × g for 15 min at 4°C. Biochemical tests were detected with Cobas8000 automatic analyzer (Roche).

### Plasmids and transfection

The full-length ORF of human *Ace2*, *Axl* and *Furin* was PCR amplified and inserted into pcDNA3.1 vector respectively (G4WT). Their corresponding G4-mutant (G4MUT) plasmids were generated by site-directed mutagenesis, in which guanines in each G-tracts of PQSs were substituted with adenines. Additionally, the wild type or RG4 region mutant 5’UTR of *Axl* was inserted before its ORF, since the PQS of *Axl* is localized in its 5’UTR.

For plasmids transfection, cells were seeded into 6- or 12-well plates and transfected with indicated plasmids via Attractene (QIAGEN, 1051563) following the manufacturer’s protocol.

### SARS-CoV-2 pseudovirus entry for cell culture

The *in vitro* SARS-CoV-2 pseudovirus assay was performed according to our previous study [17]. In brief, cells were cultured in 96-well plates, and inoculated with pseudovirus at a multiplicity of infection (MOI) of 1 at 60–80% confluence. For drug intervention, PDS (MCE, HY-15176A), TPT and BBM at indicated dose were added to cells respectively 5 hrs before transduction. For plasmids transfection, cells were transfected with indicated plasmids 24 hrs before transduction. After 16 hrs post transduction, the culture medium was replaced with a fresh medium. Transduction efficiency was then quantified 48 hrs post transduction by measuring luciferase activity in cell lysates using the ONE-Glo Luciferase Assay (E6120, Promega, USA) according to the manufacturer’s instructions.

### Cell ability assay

1.5 × 10^4^ cells were cultured in 96-well plates and stimulated with PDS, TPT and BBM compounds at the indicated dose for 24 hrs. The cell ability was assessed using the CellTiter 96 AQ_ueous_ Non-radioactive Cell Proliferation Assay (Promega, G5421).

### Western blot

Cells were washed once in ice-cold PBS and harvested using RIPA buffer (ThermoFisher, 89901) with protease inhibitors (Roche, 11873580001). Cell lysis was performed on ice for 30 min, and samples were centrifuged. Then supernatants were denatured at 100°C for 10 min with SDS sample buffer, separated on SDS-PAGE and transferred to PVDF membranes (GE, A10122278). The membrane was blocked in 5% non-fat milk, incubated with primary and HRP-conjugated secondary antibodies, and then developed using Pierce ECL Western Blotting Substrate (ThermoFisher, 34076).

### RNA extraction and QRT-PCR

Total RNAs were harvested using Tri-Reagent (MRC, TR118) and converted to cDNA using M-MLV Reverse Transcriptase (ThermoFisher, 28025021) as described previously [61]. QRT-PCR was performed using QuantiNova SYBR Green PCR Kit (QIAGEN, 208052) and fold-inductions of target mRNA (S5 Table) levels were calculated using 2^–ΔΔCt^ taking mock non-stimulated readings as the basal level sample and *Gapdh* as the control.

### CD spectrum measurement

CD spectrum was performed according to our previous study [28]. In brief, RNAs (S6 Table) were prepared at a final concentration of 5 μM in 10 mM Tris-HCl (pH 7.5) buffer containing 150 mM KCl or LiCl, heated at 95°C for 5 min, and then gradually cooled down to 4°C. The spectra were recorded from 220 to 320 nm at a 1 nm interval by Chirascan-Plus CD Spectrometer (Applied Photophysics).

### Fluorescence emission spectrum measurement

2 μM RNAs (S6 Table) were folded in 150 mM KCl or LiCl as described above and added with 2 μM and NMM (Frontier Science, NMM580) or ThT (MCE, HY-D0218). Then samples were excited at 393 nm or 425 nm for NMM and ThT, respectively, and measured from 550–700 nm for NMM and 450–650 nm for ThT using Hybrid Multi-Mode Reader (Bioteck).

### MST measurement

CY5-labelled RNAs (S6 Table) were folded in 150 mM KCl as described above. 160 μM TPT and 80 μM BBM were prepared in 150 mM KCl respectively, and a series of 16–1:1 dilution for each ligand were prepared in the same buffer. Then RNA samples and ligands were mixed in a 1:1 radio, which led to a final ligand concentration in the μM to nM range. The mixtures were loaded into MO-K003 Monolith NT.115 hydrophobic capillaries (Nano Temper) and measured at 20% LED and medium MST power.

### Molecular docking

To predict the potential interaction of drug candidates and RG4, the CDOCKER module in Discovery Studio (Accelrys Software Inc.) was applied to our molecular docking algorithm. The three-dimensional (3D) structures of human telomeric RNA (TERRA) quadruplex (PDB code: 2M18 and 2KBP) were selected from Protein Data Bank (PDB) (https://www.rcsb.org/). The 3D structure of Berbamine (CID: 275182), Berberine (CID: 2353), Palmatine (CID: 19009), Tetrandrine (CID: 73038) and Topotecan (CID: 60700) were downloaded from The PubChemProject (https://pubchem.ncbi.nlm.nih.gov/). The top affinity scored hits were subjected to visual inspection.

### Sequence conservation analyses

Sequences of host factors were retrieved from the National Center for Biotechnology Information (NCBI) Nucleotide (https://www.ncbi.nlm.nih.gov/nuccore). Sequences of ancestral SARS-CoV-2 (Accession: MN908947.3) and nine variants, namely B.1.1.7 (Accession: OV054768), B.1.351 (Accession: OM463433.1), P.1 (Accession: MZ477859), B.1.617.2 (Accession: OK091006), B.1.1.529 (Accession: OM287553.1), XBB (Accession: OP954981), BQ.1 (Accession: OP954976), BA.5 (Accession: OP955457), and BA.2.75.2 (Accession: OP955194), were derived from NCBI Virus (https://www.ncbi.nlm.nih.gov/labs/virus/vssi/#/). The sequence alignments of PQSs were performed by using the WebLogo software (https://weblogo.threeplusone.com/).

### Statistical analysis

All data represented at least three independent experiments and were shown as mean ± SEM. GraphPad Prism 9 was used for graph preparation and statistical analysis. Two-tailed unpaired Student’s t test was used to determine the difference between two independent groups unless otherwise indicated, and p < 0.05 was considered significant.

## Supporting information

S1 Fig Identification of RG4s in SARS-CoV-2 host factors. (A) RG4 potential of all experimental verified and bioinformatic predicted SARS-CoV-2 host factors in this study. PQSs are identified by QGRS-mapper. (B) Graphical representations of RG4 sequence conservation of PQSs in *Ace2*, *Dpp4*, *Furin*, *Golga7* and *Zdhhc5*. (C, D) ThT (C) and NMM (D) fluorescence emission spectra for PQS-1682 (first panel), PQS-2302 (second panel), PQS-91 (third panel), PQS-1234 (fourth panel) and PQS-1276 (fifth panel) under KCl or LiCl conditions. (E) The WT and RG4 mutant (MUT) sequences of PQS-2302, PQS-91, PQS-1234, and PQS-1276 RNA used for RG4 characterization. (F) Schematic representation of RG4 structures in PQS-2302, PQS-91, PQS-1234, and PQS-1276.
(TIF)

S2 Fig TPT and BBM inhibit expressions of SARS-CoV-2 host factors at post-transcriptional level. (A) mRNA levels of *Ace2*, *Axl*, *Furin* or *Tmprss2* in H1299 cells transfected with ACE2/AXL/FURIN-G4WT or their corresponding G4MUT plasmids. (B) Replicated images of western blot for Fig 3B–3E. (C) mRNA levels of *Tmprss2* in H1299 cells transfected with TMPRSS2-G4WT (left panel) or -G4MUT (right panel) plasmids in the presence of TPT or BBM. (D) mRNA levels of *Tmprss2* in H1299 cells treated with TPT or BBM. Data are represented as mean ± SEM, n = 3. *p < 0.05, ns, not significance (Two-tailed Student’s t test).
(TIF)

S3 Fig Toxicity analysis of TPT and BBM on VSV-SARS-2-S-luc-infected mice. (A) Sequence conservation of PQSs in *Axl* and *Furin* between human and mouse. The bold letters represent the G-tracts. (B) The hematological parameters of ALT (left panel), AST (middle panel), and CKMB (right panel) from the peripheral blood of the mice treated with TPT or BBM. (C) Representative H&E staining of lungs, kidneys, livers and hearts from mice treated with TPT or BBM. Scale bars, 100 μm. Data are represented as mean ± SEM, n = 6.
(TIF)

S4 Fig Sequence alignments of PQSs from the ancestral SARS-CoV-2 and nine VOCs. The sequence alignments of PQSs from the ancestral SARS-CoV-2 and nine variants, Alpha (B.1.1.7), Beta (B.1.351), Gamma (P.1), Delta (B.1.617.2), and Omicron (B.1.1.529, XBB, BQ.1, BA.5 and BA.2.75.2) derived from the National Center for Biotechnology Information (NCBI) using WebLogo software.
(TIF)

S1 Table Putative RG4s in SARS-CoV-2 host factors. (DOCX)

S2 Table Summary of G4 stabilizer candidates. (DOCX)

S3 Table Docking scores between RG4s and ligands. (DOCX)

S4 Table Putative RG4s in mouse *Axl* and *Furin*. (DOCX)

S5 Table Primers used in this study. (DOCX)

S6 Table Sequences of oligomers used in this study. (DOCX)
