# Supplementary figures and images for: Targeting RNA G-quadruplex with repurposed drugs blocks SARS-CoV-2 entry
Source: PLoS Pathog. 2023 Jan 26;19(1):e1011131. doi: 10.1371/journal.ppat.1011131 (PMC9904497; doi:10.1371/journal.ppat.1011131)

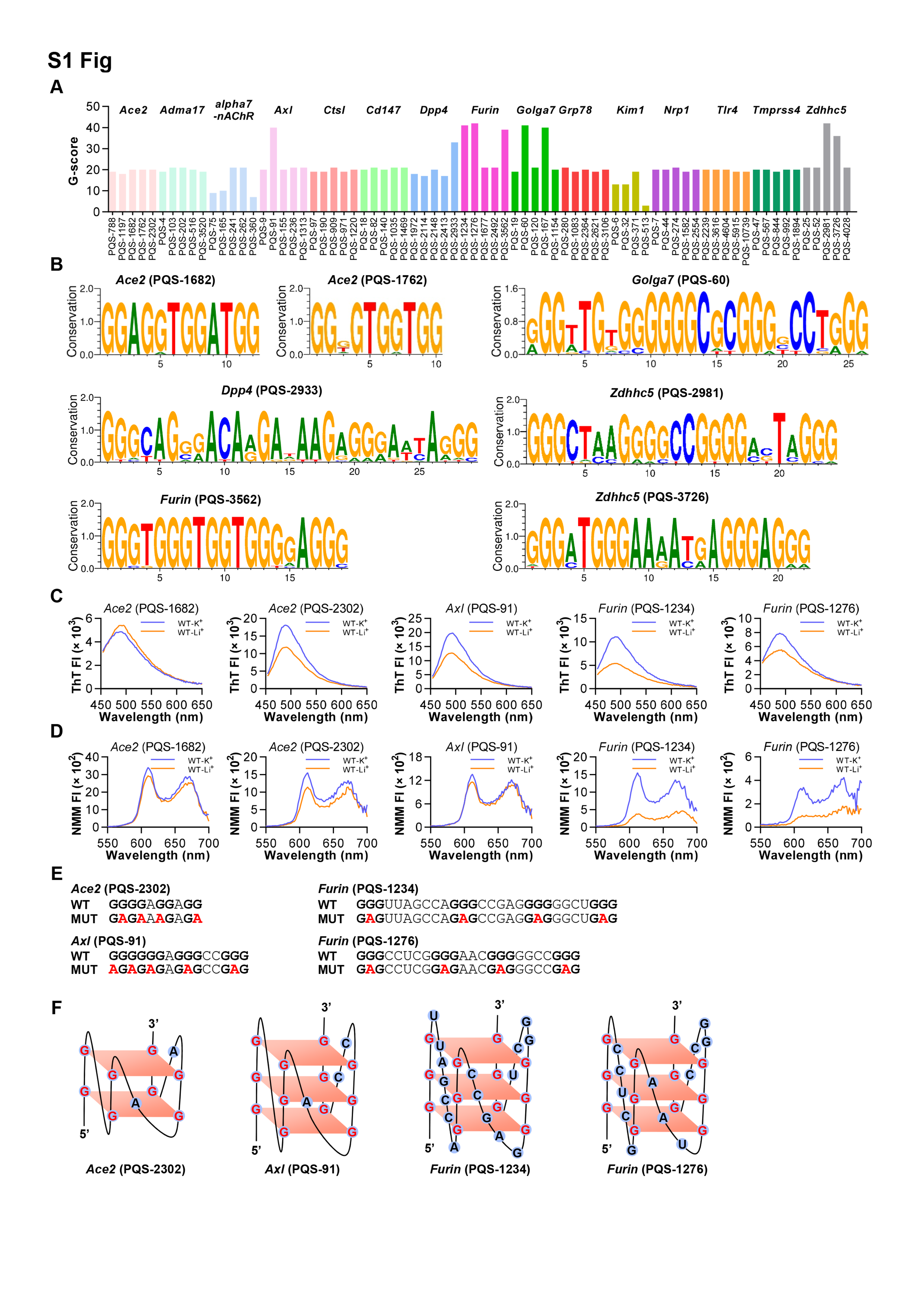

Supplement: S1 Fig — (A) RG4 potential of all experimental verified and bioinformatic predicted SARS-CoV-2 host factors in this study. PQSs are identified by QGRS-mapper. (B) Graphical representations of RG4 sequence conservation of PQSs in Ace2, Dpp4, Furin, Golga7 and Zdhhc5. (C, D) ThT (C) and NMM (D) fluorescence emission spectra for PQS-1682 (first panel), PQS-2302 (second panel), PQS-91 (third panel), PQS-1234 (fourth panel) and PQS-1276 (fifth panel) under KCl or LiCl conditions. (E) The WT and RG4 mutant (MUT) sequences of PQS-2302, PQS-91, PQS-1234, and PQS-1276 RNA used for RG4 characterization. (F) Schematic representation of RG4 structures in PQS-2302, PQS-91, PQS-1234, and PQS-1276. (TIF) [file ppat.1011131.s001.tif]

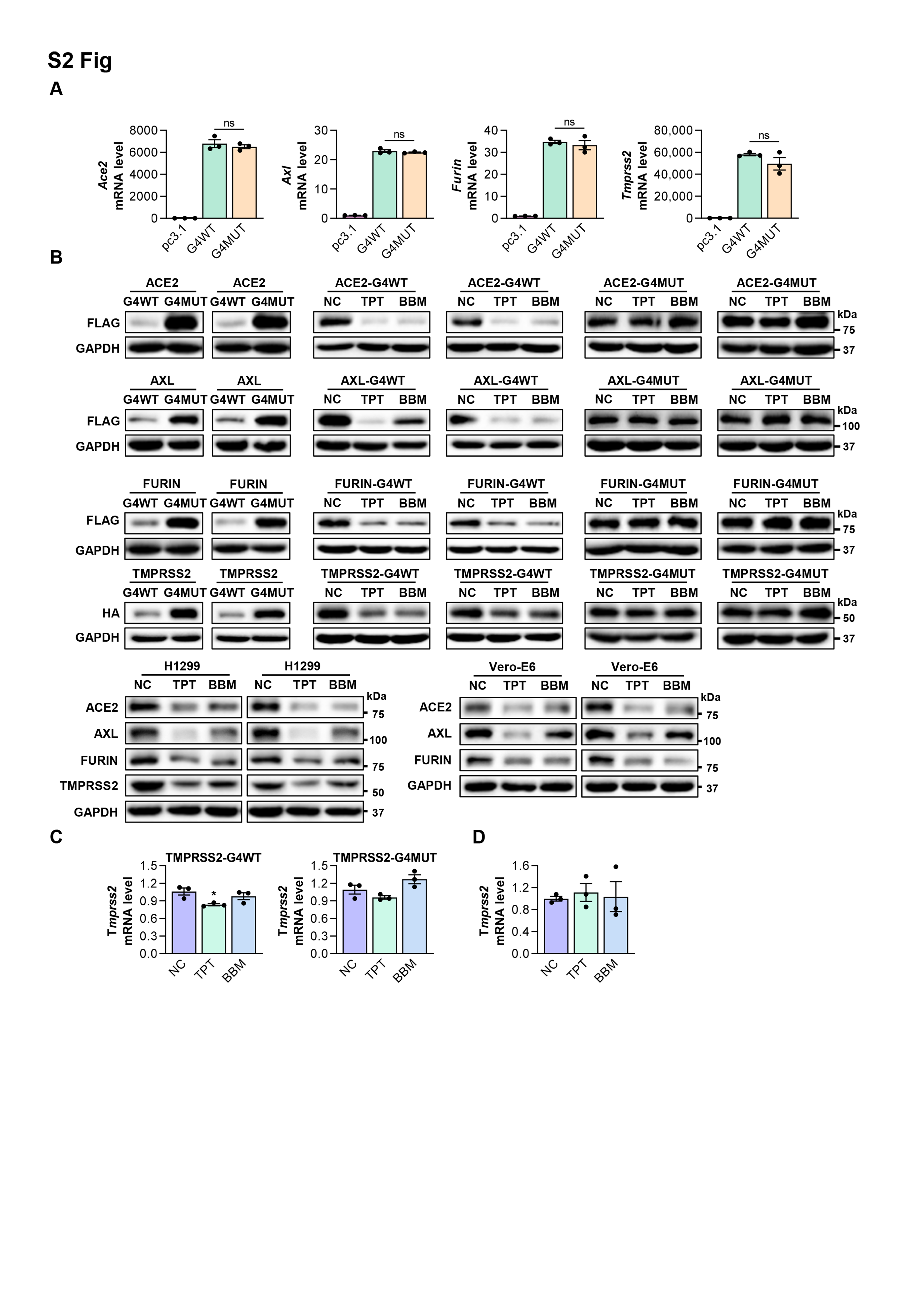

Supplement: S2 Fig — (A) mRNA levels of Ace2, Axl, Furin or Tmprss2 in H1299 cells transfected with ACE2/AXL/FURIN-G4WT or their corresponding G4MUT plasmids. (B) Replicated images of western blot for Fig 3B–3E. (C) mRNA levels of Tmprss2 in H1299 cells transfected with TMPRSS2-G4WT (left panel) or -G4MUT (right panel) plasmids in the presence of TPT or BBM. (D) mRNA levels of Tmprss2 in H1299 cells treated with TPT or BBM. Data are represented as mean ± SEM, n = 3. *p < 0.05, ns, not significance (Two-tailed Student’s t test). (TIF) [file ppat.1011131.s002.tif]

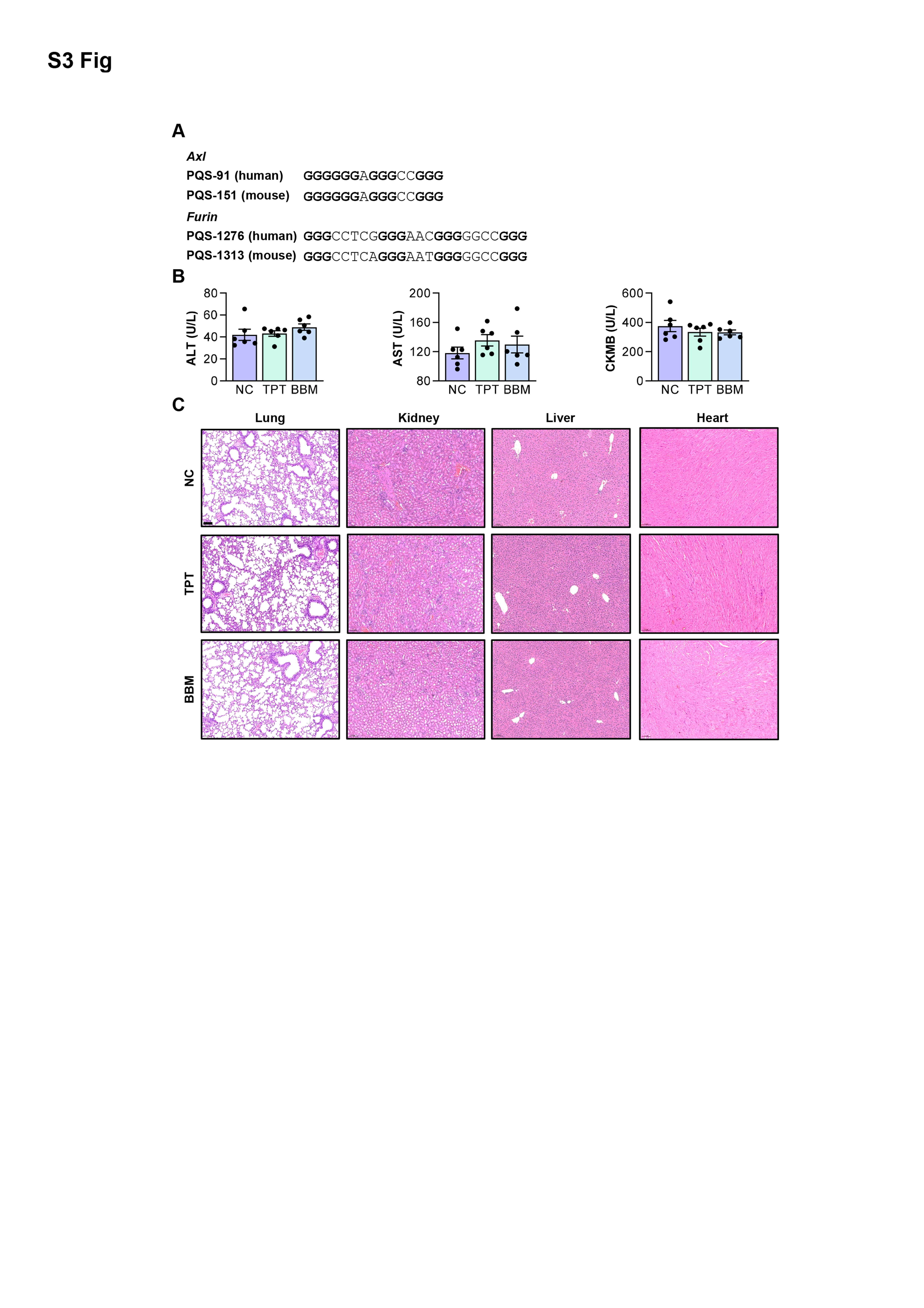

Supplement: S3 Fig — (A) Sequence conservation of PQSs in Axl and Furin between human and mouse. The bold letters represent the G-tracts. (B) The hematological parameters of ALT (left panel), AST (middle panel), and CKMB (right panel) from the peripheral blood of the mice treated with TPT or BBM. (C) Representative H&E staining of lungs, kidneys, livers and hearts from mice treated with TPT or BBM. Scale bars, 100 μm. Data are represented as mean ± SEM, n = 6. (TIF) [file ppat.1011131.s003.tif]

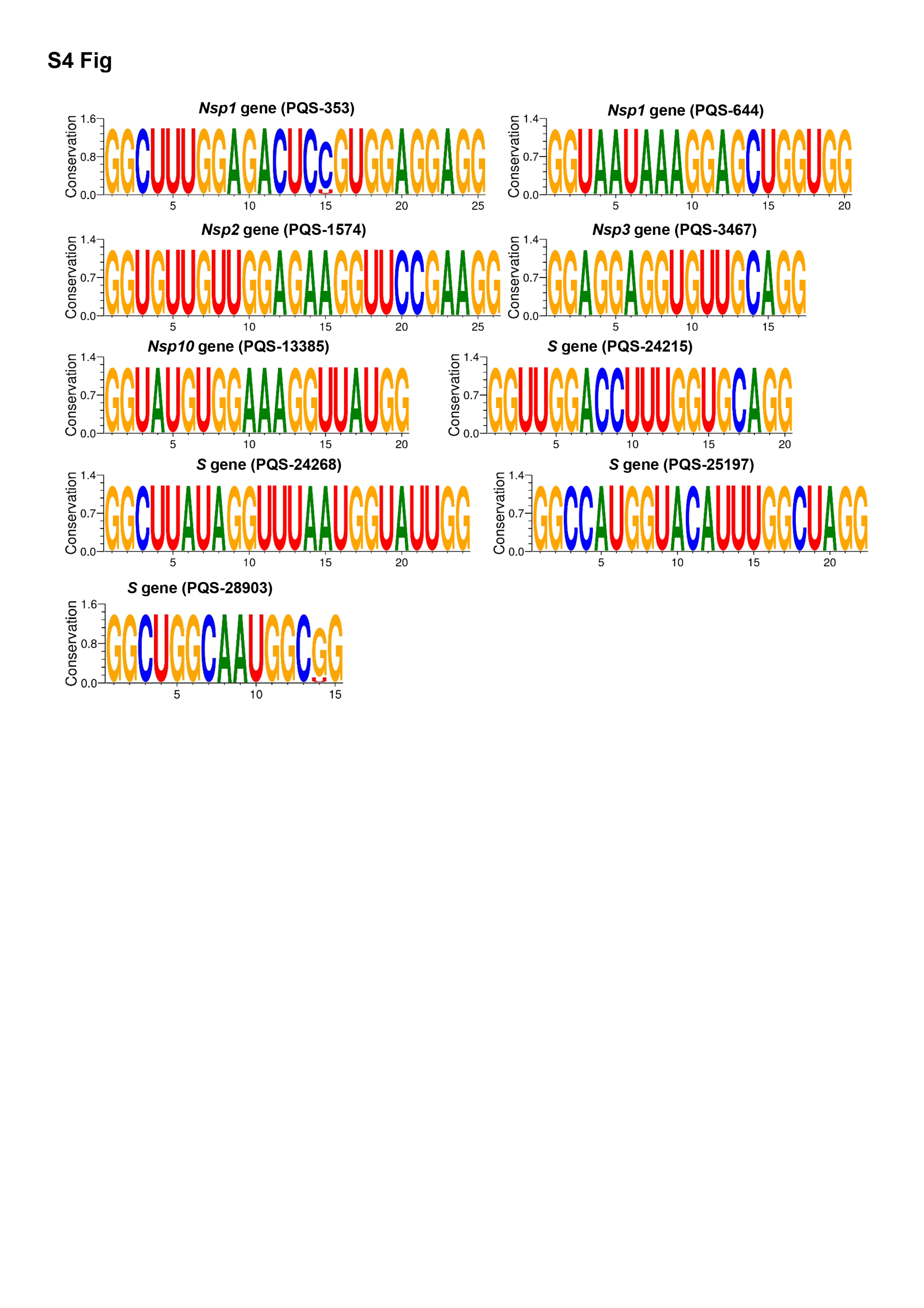

Supplement: S4 Fig — The sequence alignments of PQSs from the ancestral SARS-CoV-2 and nine variants, Alpha (B.1.1.7), Beta (B.1.351), Gamma (P.1), Delta (B.1.617.2), and Omicron (B.1.1.529, XBB, BQ.1, BA.5 and BA.2.75.2) derived from the National Center for Biotechnology Information (NCBI) using WebLogo software. (TIF) [file ppat.1011131.s004.tif]
