# Supplementary material for: Targeting RNA G-quadruplex with repurposed drugs blocks SARS-CoV-2 entry
Source: PLoS Pathog. 2023 Jan 26;19(1):e1011131. doi: 10.1371/journal.ppat.1011131 (PMC9904497; doi:10.1371/journal.ppat.1011131)
Supplement: S1 Table — (DOCX) [file ppat.1011131.s005.docx]

**S1 Table. Putative RG4s in SARS-CoV-2 host factors.**

| Gene | Site | G-score | Putative RG4 sequence |
| --- | --- | --- | --- |
| *Ace2* | 788 | 19 | GGCTCTGGGCTTGGGAAAGCTGG |
|  | 1197 | 18 | GGTGGACCAGGCCTGG |
|  | 1682 | 20 | GGAGGTGGATGG |
|  | 1762 | 20 | GGGGTGGTGG |
|  | 2302 | 20 | GGGGAGGAGG |
| *Adam17* | 4 | 19 | GGCGGCCGGAAGCTGGCTGAGCCGG |
|  | 103 | 21 | GGGAAAAGAGGATTGAGGGGCTAGGCCGG |
|  | 202 | 21 | GGAGTCGGTAGCGGGGCCGG |
|  | 516 | 20 | GGTGGTGGATGG |
|  | 3520 | 19 | GGCTGGGAGTGGTGG |
| *alpha7-nAChR* | 75 | 9 | GGCCTTCCTGTCCCGCGGGAAGCGGCAGG |
|  | 165 | 10 | GGCACCCTCAGATGGCCAGACTCCTGGGG |
|  | 241 | 21 | GGATCAGGTGGAGGTGCTGG |
|  | 262 | 21 | GGAGGAGGTAGTGGAAGAGG |
|  | 360 | 7 | GGGGAAAACAACTGCAGAGGATGGG |
| *Axl* | 9 | 20 | GGCTGCTGGGCAGAGCCGGTGGCAAGGG |
|  | 91 | 40 | GGGGGGAGGGCCGGG |
|  | 155 | 20 | GGAAAGTTTGGCACCCATGGCGTGGCGG |
|  | 236 | 21 | GGCGTGCATGGCCCCCAGGGGCACGCAGG |
|  | 1313 | 21 | GGACATAGGGCTAAGGCAAGAGG |
| *Ctsl* | 97 | 19 | GGCCGGACAGGGACTGG |
|  | 190 | 19 | GGTGGGGAAGCCGGGGAGGG |
|  | 909 | 21 | GGGCCTCAAGGCAATGAAGGCTGCAATGG |
|  | 971 | 19 | GGATAATGGAGGCCTGGACTCTGAGG |
|  | 1220 | 20 | GGATCATGGTGTGCTGGTGGTTGG |
| *Cd147* | 18 | 20 | GGACCGGCGAGGAATAGG |
|  | 82 | 21 | GGCACCCACGGAGCCTCCGGGGCTGCCGG |
|  | 140 | 20 | GGTGGGTGGGGGG |
|  | 1035 | 21 | GGCTGAGGTGCTGGTGCTGG |
|  | 1469 | 21 | GGCCGGGTGGGCGG |
| *Dpp4* | 1972 | 18 | GGCAGAGGAAGTGGTTACCAAGG |
|  | 2114 | 17 | GGGGCTGGTCATATGGAGGG |
|  | 2148 | 20 | GGTCCTGGGATCGGGAAGTGG |
|  | 2413 | 17 | GGAGTGGATTTCCAGGCAATGTGG |
|  | 2933 | 33 | GGGCAGGGACAGGATAAGAGGGATTAGGG |
| *Furin* | 1234 | 41 | GGGTTAGCCAGGGCCGAGGGGGGCTGGG |
|  | 1276 | 42 | GGGCCTCGGGGAACGGGGGCCGGG |
|  | 1677 | 21 | GGCTACGGGCTTTTGGACGCAGG |
|  | 2492 | 21 | GGAGGTGGAGG |
|  | 3562 | 39 | GGGTGGGTGGTGGGGAGGG |
| *Golga7* | 19 | 19 | GGAGGGCAGAGGAGGCGG |
|  | 60 | 41 | GGGTTGTGGGGGGCGCGGGGCCTGGG |
|  | 120 | 21 | GGTGAAGGGGAGCGGGGCAGAGG |

**S1 Table** **(Continued)**

| Gene | Site | G-score | Putative RG4 sequence |
| --- | --- | --- | --- |
| *Golga7* | 167 | 40 | GGGTGGGGGCGAGGGGCTGGCGGG |
|  | 1154 | 20 | GGTGAGGGTTGGCTGTGG |
| *Grp78* | 280 | 21 | GGACAAGAAGGAGGACGTGGGCACGGTGG |
|  | 1083 | 19 | GGCGCGAGGTAGAAAAGGCCAAACGG |
|  | 2364 | 20 | GGTGGGGGGG |
|  | 2621 | 19 | GGAGTATTTGGTATGTTGGGATAAGG |
|  | 3106 | 20 | GGTACTGGGGTGGGATCTTGG |
| *Kim1* | 6 | 13 | GGAGTCAGTTTGGCGGTTATGTGTGG |
|  | 32 | 13 | GGAAGAAGCTGGGAAGTCAGGGG |
|  | 371 | 19 | GGTGGAGAGGCAGG |
|  | 513 | 3 | GGAAGGACACACGCTATAAGCTATTGGGGG |
| *Nrp1* | 7 | 20 | GGCCCGGGCAGTGGCTCCTGG |
|  | 44 | 20 | GGAAAAGGGAGAGGAAGCCGG |
|  | 274 | 21 | GGAGAAGGGAGAATGGAGAGGGG |
|  | 1582 | 19 | GGAATGTTGGGTATGGTGTCTGG |
|  | 2554 | 20 | GGACACCAAGGTGACCACTGGAAGGAAGGG |
| *Tlr4* | 2239 | 20 | GGATGAGGACTGGGTAAGG |
|  | 3616 | 20 | GGGCTAGAGGCAGGAAGGAAGTGGG |
|  | 4604 | 20 | GGTTCCTAGGGAAAAGGAGGAAGG |
|  | 5915 | 19 | GGCAGGTGAGGTGG |
|  | 10739 | 19 | GGTAGTGAGGTTTAGGATTCAGG |
| *Tmprss4* | 47 | 20 | GGATGCTGGGCGTGAGGGACCAAGG |
|  | 567 | 20 | GGTGCTGGACTCGGCCACAGG |
|  | 844 | 19 | GGTGTGGAGGAGG |
|  | 992 | 20 | GGAAGGTGCGGGCAGG |
|  | 1894 | 20 | GGAGAGGAGAAGGAAAGG |
| *Zdhhc5* | 25 | 21 | GGAGGCGGCGG |
|  | 52 | 21 | GGTGACGGGCACCGGGCTCGCGG |
|  | 2981 | 42 | GGGCTAAGGGGCCGGGGAGTAGGG |
|  | 3726 | 36 | GGGATGGGAAAATGAGGGAGGG |
|  | 4028 | 21 | GGTGGTGGTGG |

Note: G-tracts in the putative RG4 region are underlined.
