# Supplementary material for: Targeting RNA G-quadruplex with repurposed drugs blocks SARS-CoV-2 entry
Source: PLoS Pathog. 2023 Jan 26;19(1):e1011131. doi: 10.1371/journal.ppat.1011131 (PMC9904497; doi:10.1371/journal.ppat.1011131)
Supplement: S2 Table — (DOCX) [file ppat.1011131.s006.docx]

**S2 Table. Summary of G4 stabilizer candidates.**

| Ligand | G4 Type | Indication | Clinical Approved | |
| --- | --- | --- | --- | --- |
| Adriamycin | DG4 | Cancer | | FDA approved |
| APTO-253 | DG4 |  | |  |
| Amidoxime | RG4 |  | |  |
| Ant1,5 | DG4 |  | |  |
| AQ1 | DG4 |  | |  |
| Benzo[a]phenoxazines | DG4 |  | |  |
| Benzofuran | DG4 |  | |  |
| Benzoselenoxanthene | DG4 |  | |  |
| Berberine | DG4 | Infectious gastroenteritis | | NMPA approved |
| Berbamine | DG4 | Leukopenia | | NMPA approved |
| Bleomycin | DG4 | Cancer | | FDA/NMPA approved |
| BRACO-19 | DG4/RG4 |  | |  |
| Carbazole | DG4 |  | |  |
| Carboxypyridostatin | RG4 |  | |  |
| Cepharanthine | DG4 |  | |  |
| c-exNDI | DG4 |  | |  |
| CX-3543 | DG4 | Advanced solid tumor | | Phase I (NCT00955786) |
|  |  | Neuroendocrine tumor | | Phase II (NCT00780663) |
| CX-5461 | DG4 | Advanced solid tumor | | Phase I (NCT04890613) |
|  |  | Cancer | | Phase I (NCT02719977) |
| C3 | DG4 |  | |  |
| CM03 | DG4 |  | |  |
| CORON | DG4 |  | |  |
| DIZ-3 | DG4 |  | |  |
| DTE | DG4 |  | |  |
| EMICORON | BG4 |  | |  |
| Epirubicin | DG4 | Cancer | | FDA /NMPA approved |
| Fangchinoline | DG4 |  | |  |
| Furopyridazinones | DG4 |  | |  |
| GQC-05 | DG4 |  | |  |
| GSA1129 | DG4 |  | |  |
| dGTC365 | DG4 |  | |  |
| Indoloquinoline | DG4 |  | |  |
| Isoalloxazines | DG4 |  | |  |
| IZCZ-3 | DG4 |  | |  |
| Jatrorrhizine | DG4/RG4 |  | |  |
| L1H1-7OTD | DG4 |  | |  |
| Liensinine | DG4 |  | |  |
| Mitoxantrone | DG4 | Rhabdomyosarcoma | | FDA/NMPA approved |
|  |  | Advanced solid tumor | | Phase I (NCT04921878) |

**S2 Table (Continued)**

| Ligand | G4 Type | Indication | Clinical Approved |
| --- | --- | --- | --- |
| MMQ3 | DG4 |  |  |
| m-TMPipEOPP | DG4 |  |  |
| Naphthalene diimides | DG4 |  |  |
| Nitidine | DG4 |  |  |
| NMM | DG4/RG4 |  |  |
| Palmatine | DG4/RG4 | Headache, Visceralgia | NMPA approved |
| PBP2 | DG4 |  |  |
| PhenDC3 | DG4/RG4 |  |  |
| PhenDH2 | RG4 |  |  |
| PIPER | BG4 |  |  |
| PM2 | DG4 |  |  |
| PyDH2 | RG4 |  |  |
| Pyridostatin | DG4/RG4 |  |  |
| QN-1 | DG4 |  |  |
| Quercetin | DG4 | COVID-19 | Phase III (NCT04578158) |
|  |  | Chronic hepatitis C | Phase I (NCT01438320) |
| Quinazoline | DG4 |  |  |
| RHPS4 | DG4 |  |  |
| Ru-Schiff | DG4 |  |  |
| Sanquinarine | DG4/RG4 |  |  |
| Se2SAP | DG4 |  |  |
| Schizocommunins | DG4 |  |  |
| Stiff-stilbenes | DG4 |  |  |
| S4-5 | DG4 |  |  |
| Telomestatin | DG4 |  |  |
| Tetrandrine | DG4/RG4 | Neuralgia, Silicosis | NMPA approved |
| TH3 | DG4 |  |  |
| TMPyP4 | DG4/RG4 |  |  |
| Topotecan | DG4 | Cancer | FDA approved |
|  |  | COVID-19 | Phase I (NCT05083000) |
| (Zn)TCPPSpm4 | DG4 |  |  |
| 20A | DG4 |  |  |
| 360A | DG4/RG4 |  |  |
| Λ-Ru | DG4 |  |  |
