# Supplementary material for: Targeting RNA G-quadruplex with repurposed drugs blocks SARS-CoV-2 entry
Source: PLoS Pathog. 2023 Jan 26;19(1):e1011131. doi: 10.1371/journal.ppat.1011131 (PMC9904497; doi:10.1371/journal.ppat.1011131)
Supplement: S3 Table — (DOCX) [file ppat.1011131.s007.docx]

**S3 Table. Docking scores between RG4s and ligands.**

| Ligands name | 2KBP | | | | 2M18 | | |
| --- | --- | --- | --- | --- | --- | --- | --- |
|  | **Absolute energy** | **Relative energy** | **Libdock score** | | **Absolute energy** | **Relative energy** | **Libdock score** |
| Berbamine | 129.6040 | 11.3495 | 81.9916 | 128.0580 | | 9.8035 | 96.4966 |
| Berberine | 114.5510 | 11.3899 | 78.9045 | 103.5560 | | 0.3956 | 84.8153 |
| Palmatine | 86.7147 | 7.3497 | 68.1385 | 88.0391 | | 8.6741 | 111.9330 |
| Tetrandrine | 128.1520 | 8.4178 | 13.8423 | 119.7910 | | 0.0565 | 57.3535 |
| Topotecan | 80.3437 | 7.5863 | 102.6390 | 81.3504 | | 8.5930 | 130.4940 |
