# Supplementary material for: Targeting RNA G-quadruplex with repurposed drugs blocks SARS-CoV-2 entry
Source: PLoS Pathog. 2023 Jan 26;19(1):e1011131. doi: 10.1371/journal.ppat.1011131 (PMC9904497; doi:10.1371/journal.ppat.1011131)
Supplement: S4 Table — (DOCX) [file ppat.1011131.s008.docx]

**S4 Table. Putative RG4s in mouse *Axl* and *Furin*.**

| Gene | Site | G-score | Putative RG4 sequence |
| --- | --- | --- | --- |
| *mAxl* | 6 | 35 | GGGAGCCAGGGGCGGGGAAAGAAGTCTGGG |
|  | 151 | 40 | GGGGGGAGGGCCGGG |
|  | 244 | 20 | GGTGTCTGGAGGATGGGCAGGG |
|  | 373 | 20 | GGTGCCAGAGGACTCACGGGGACACTTCGG |
|  | 954 | 20 | GGAGCTAGAGGTAGCTTGGACCCCTGG |
| *mFurin* | 1269 | 35 | GGGAGTTAGCCAGGGCCGAGGAGGGCTGGG |
|  | 1313 | 40 | GGGCCTCAGGGAATGGGGGCCGGG |
|  | 1714 | 21 | GGCTACGGGCTGTTGGATGCAGG |
|  | 1818 | 21 | GGACATCGGCAAACGGCTAGAGG |
|  | 3548 | 21 | GGGTGGGTGGTGGG |

Note: G-tracts in the putative RG4 region are underlined.
