# Supplementary material for: Targeting RNA G-quadruplex with repurposed drugs blocks SARS-CoV-2 entry
Source: PLoS Pathog. 2023 Jan 26;19(1):e1011131. doi: 10.1371/journal.ppat.1011131 (PMC9904497; doi:10.1371/journal.ppat.1011131)
Supplement: S5 Table — (DOCX) [file ppat.1011131.s009.docx]

**S5 Table. Primers used in this study.**

| Genes | Primers (from 5’-3’) |
| --- | --- |
| Human *Ace2* | F: CGAAGCCGAAGACCTGTTCTA |
|  | R: GGGCAAGTGTGGACTGTTCC |
| Human *Axl* | F: GTGGGCAACCCAGGGAATATC |
|  | R: GTACTGTCCCGTGTCGGAAAG |
| Mouse *Axl* | F: ATGGCCGACATTGCCAGTG |
|  | R: CGGTAGTAATCCCCGTTGTAGA |
| Human *Furin* | F: TCGGGGACTATTACCACTTCTG |
|  | R: CCAGCCACTGTACTTGAGGC |
| Mouse *Furin* | F: TCGGTGACTATTACCACTTCTGG |
|  | R: CTCCTGATACACGTCCCTCTT |
| Human *Tmprss2* | F: GCAGTGGTTTCTTTACGCTGT |
|  | R: CCGCAAATGCCGTCCAATG |
| Mouse *Tmprss2* | F: ATGCTCCGAGGATTACAACGC |
|  | R: CGAGGGCTAAACACAGCGATT |
| Human *Gapdh* | F: ACAGCCTCAAGATCATCAGCAA |
|  | R: ACCACTGACACGTTGGCAGT |
| Mouse *Gapdh* | F: AGGTCGGTGTGAACGGATTTG |
|  | R: TGTAGACCATGTAGTTGAGGTCA |
| ACE2-G4WT | F: ACAAGGATGACGATGACAAGATGTCAAGCTCTTCCTGGCTCC |
|  | R: CAGCGGGTTTAAACGGGCCCCTAAAAGGAGGTCTGAACATCATCAG |
| ACE2-G4MUT | F: GAAGAGAAAGATGTGCGAGTGGCTAATTTGAAA |
|  | R: CGCACATCTTTCTCTTCAAAAAGAATCATCTGATTTTTTACTTTTAA |
| AXL-G4WT | F: ACAAGGATGACGATGACAAGGAGAAGGCGGCTGCTGGG |
|  | R: CAGCGGGTTTAAACGGGCCCTCAGGCACCATCCTCCTGC |
| AXL-G4MUT | F: TGAGAGAAGAGCCGAGAACAGCCCGGCCCTGCCC |
|  | R: TCTCGGCTCTTCTCTCAGCTCCAGGCTCCCCGGA |
| FURIN-G4WT | F: ACAAGGATGACGATGACAAGATGGAGCTGAGGCCCTGG |
|  | R: CAGCGGGTTTAAACGGGCCCCTGGTTTGCATAGTCTGCACGT |
| FURIN-G4MUT | F: GAAACGGAGCCGAGAACATGACAGCTGCAACTGCGA |
|  | R: TTCTCGGCCTCCGTTTCCTGAGGCCCAGACAAAGATGGAGCCCAGCC |
