# Supplementary material for: Targeting RNA G-quadruplex with repurposed drugs blocks SARS-CoV-2 entry
Source: PLoS Pathog. 2023 Jan 26;19(1):e1011131. doi: 10.1371/journal.ppat.1011131 (PMC9904497; doi:10.1371/journal.ppat.1011131)
Supplement: S6 Table — (DOCX) [file ppat.1011131.s010.docx]

**S6 Table. Sequences of oligomers used in this study.**

| Name | Sequence (from 5’-3’) |
| --- | --- |
| Human *Ace2* | |
| PQS-1682-WT | GGAGGUGGAUGG |
| PQS-1762-WT | GGGGUGGUGG |
| PQS-2302-WT | GGGGAGGAGG |
| PQS-2302-MUT | GAGAAAGAGA |
| PQS-2302-WT-CY5 | CY5-GGGGAGGAGG |
| PQS-2302-MUT-CY5 | CY5-GAGAAAGAGA |
| Human *Axl* | |
| PQS-91-WT  PQS-91-MUT | GGGGGGAGGGCCGGG  AGAGAGAGAGCCGAG |
| Human *Dpp4* | |
| PQS-2933-WT | GGGCAGGGACAGGAUAAGAGGGAUUAGGG |
| Human *Furin* | |
| PQS-1234-WT | GGGUUAGCCAGGGCCGAGGGGGGCUGGG |
| PQS-1234-MUT | GAGUUAGCCAGAGCCGAGGAGGGCUGAG |
| PQS-1276-WT | GGGCCUCGGGGAACGGGGGCCGGG |
| PQS-1276-MUT | GAGCCUCGAGGAACGAGGGCCGAG |
| PQS-3562-WT | GGGUGGGUGGUGGGGAGGG |
| Human *Golga7* |  |
| PQS-60-WT | GGGUUGUGGGGGGCGCGGGGCCUGGG |
| Human *TERRA* | |
| TERRA-WT-CY5 | CY5-UUAGGGUUAGGGUUAGGGUUAGGG |
| TERRA-MUT-CY5 | CY5-UUACCGUUACCGUUACCGUUACCG |
| Human *Tmprss2* | |
| PQS-675-WT-CY5 | CY5-GGGCGGGCGGCCUGCAGGGACAUGGG |
| PQS-675-MUT-CY5 | CY5-GAGCGAGCGGCCUGCAGAGACAUGAG |
| Human *Zdhhc5* | |
| PQS-2981-WT | GGGCUAAGGGGCCGGGGAGUAGGG |
| PQS-3726-WT | GGGAUGGGAAAAUGAGGGAGGG |
