# Supplementary material for: Targeting RNA G-quadruplex with repurposed drugs blocks SARS-CoV-2 entry
Source: PLoS Pathog. 2023 Jan 26;19(1):e1011131. doi: 10.1371/journal.ppat.1011131 (PMC9904497; doi:10.1371/journal.ppat.1011131)
Supplement: Attachment — Submitted filename: Response letter to Reviewers.pdf [file ppat.1011131.s011.pdf]

Dear Editors and Reviewers,

We deeply appreciate your encouraging and insightful comments/suggestions regarding our original submission. In response to these comments, we have performed additional suggested experiments and made a number of modifications to our manuscript. Below we detail these modifications with the specific comments from the reviewers (in *italic*), followed by our response (in blue). We have highlighted all changes underlined in the revised manuscript. We hope the reviewers will find this manuscript improved following these changes and more suitable for publication in ***PLOS Pathogens***.

The point-by-point responses to all of the reviewers' comments are listed below:

Reviewer 1

The manuscript by Tong et al. reports the potential of RG4-targeting drugs on SARS-CoV-2 infection. Initially, the authors screened numerous SARS-CoV-2 host factors by bioinformatic analysis to predict potential RG4s, and then characterized novel RG4s within three host factors, including Ace2, Axl and Furin by a combination of several biochemical and biophysical methods. Subsequently, the authors identified TPT and BBM, two approved clinical drugs, as RG4-stabilizing agents by in silico screening of G4 ligand library and literature searching, followed by microscale thermophoresis validation. Next, the authors performed a series of experiments to verify that RG4s inhibited the expression of ACE2, AXL and FURIN, as well as the previous known RG4-containing TMPRSS2. Importantly, the authors provided strong evidence to demonstrate that both TPT and BBM can effectively repress the expression of ACE2, AXL, FURIN, and TMPRSS2 through the RG4 sequence, and potently reduce the infection of SARS-CoV-2 pseudovirus. Finally, they confirmed the

inhibitory effect of TPT and BBM on SARS-CoV infection in mouse models in vivo.

This study not only expands the existence of RG4 in SARS-CoV-2 host factors, but also provides a novel, to my knowledge, RG4-targeting strategy for COVID-19 prevention and therapy. This manuscript is well designed, and the data are solid. Overall, this timely and novel study is of interest and importance to the pathophysiology and treatment of COVID-19, which could offer new insights to beat the ongoing pandemic.

Response: We are very grateful to the reviewer's encouraging and thoughtful comments.

Major comments

1. *It has clearly showed that the mutations of RG4 sites can increase the protein levels of exogenous host factors, including ACE2, AXL, FURIN, and TMPRSS2 (Fig. 3A). These data are critical to demonstrate that the formation of RG4 structure can repress the expression of these host factors. However, it remains unclear whether the increase in protein level is due to the difference of transfected plasmid or not. In this regard, the authors should provide the mRNA levels of these host factors after transfection.*

Response: Thank the reviewer for this crucial comment. As suggested, we have determined the effect of plasmid transfection on mRNA expression. It showed that transfection of G4WT and G4MUT plasmids into H1299 cells led to a comparable increase in *Ace2, Axl, Furin,* and *Tmprss2* mRNAs (**Figure below**), supporting a post-transcriptionally inhibitory effect of RG4 structure on host factors expression. These results are now described in **S2A Fig**.

S2A Fig

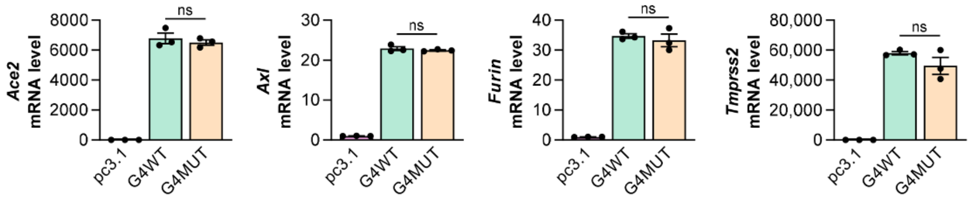

2. *Both TPT and BBM showed considerable inhibition on ACE2-G4WT expression (Fig. 3B). However, an inhibitory effect was also seen on ACE2-G4MUT expression (Fig. 3C). What is the potential explanation? It is better to perform additional experiments to strengthen this conclusion, if applicable.*

Response: We appreciate the reviewer for this critical comment. In addition to the dominant PQS-2302, PQS-1682 also has the possibility to form RG4 (**Fig. 1C** and **S1C Fig**). In the original version, only PQS-2302 was mutated in the ACE2-G4MUT plasmid, which may be responsible for the observed inhibition. In this regard, we have generated a new ACE2-G4MUT plasmid, in which both PQS-2303 and PQS-1682 were mutated with synonymous substitution (**Figure below**). Western blot analysis showed that TPT and BBM had no effect on ACE2 expression in G4MUT transfected cells (**Figure below**). Three repeated experiments were shown, as raised by **Reviewer 3, Point 1** (Red outlines indicated the representative image in the main text). These results are now described in **Fig 3A, 3D** and **S2B Fig**.

Fig 3A

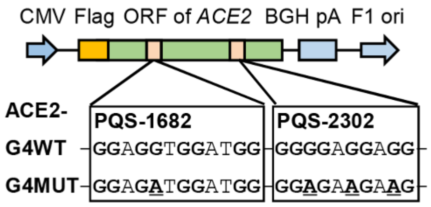

Fig 3D & S2B Fig

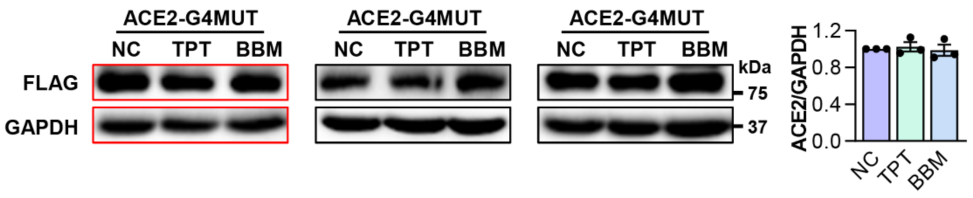

Minor comments

3. *It is better to insert S2A-C Fig to Fig. 3. It would be easier for the readers to understand the mutations.*

Response: Thank the reviewer for the suggestion. As suggested, this data have been transferred and presented as **Fig. 3A**.

4. *The authors should perform the immunohistochemical assay of certain host factors to further confirm the inhibitory effect of TPT and BBM on RG4-containing host factors.*

Response: We appreciate the reviewer for this thoughtful comment. Following the suggestion, we have performed IHC assay and further confirmed decreases in AXL, FURIN and TMPRSS2 protein level in lungs of TPT- and BBM-treated mice (**Figure below**), consistent with our western blot data (**Fig. 5E** and **5F**). These results are now described in **Fig 5G**.

Fig 5G

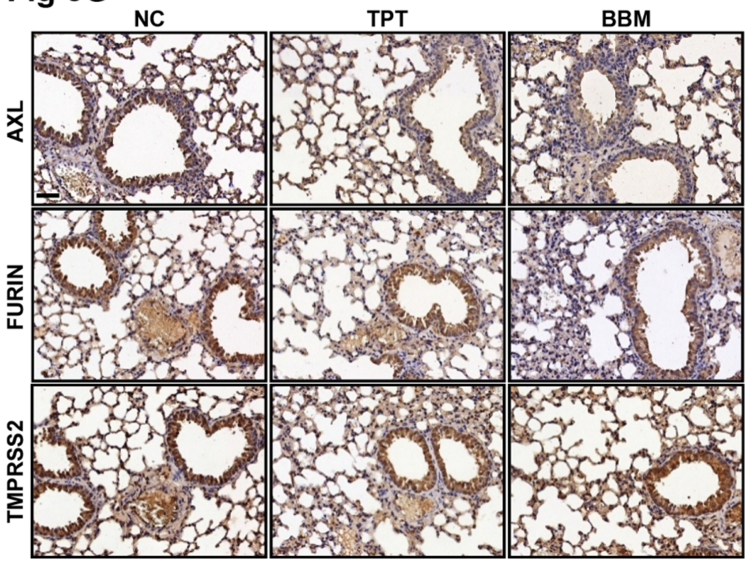

5. *Since the in vivo function of TPT and BBM were determined in mouse models, the sequence conservation of the human ACE2, AXL, FURIN RG4s in the corresponding murine mRNAs should be provided. If the*

sequence conservation is low, the authors should provide experimental data to verify the existence of RG4s within these murine host factors.

Response: Thank the reviewer for this important comment. Following the suggestion, the human and corresponding murine ACE2, AXL, FURIN RG4s have been aligned (**Figure below**).To further improve the clarity, we have also revised the main text as the follows: "Notably, the sequence of PQS-91 in human *Axl* is identical to that of PQS-151 in mouse homolog (S3A Fig)." Of note, mouse ACE2 is incapable of mediating SARS-CoV-2 entry, therefore, the sequence conservation of ACE2 RG4 was neglected in this study, which has been mentioned in the main text (**Page 13, line 11**). These results are now described in **S3A Fig**.

S3A Fig

Axl

**PQS-91 (human)**	GGGGGGAGGGCCGGG
**PQS-151 (mouse)**	GGGGGGAGGGCCGGG

Furin

**PQS-1276 (human)**	GGGCCTCGGGGAACGGGGGCCGGG
**PQS-1313 (mouse)**	GGGCCTCAGGGAATGGGGGCCGGG

6. *BBM showed a better effect on SARS-CoV-2 infection than TPT in cultured cells, but seemed to be less than TPT in mouse models. The authors should appropriately discuss this point.*

Response: Thank the reviewer for this important comment. We totally agree with the reviewer on the different effectiveness *in vitro* and *in vivo*. A possible explanation is that the concentration of TPT and BBM used in cultured cells comparatively high, which is a safety threshold without inducing cell death. While the concentration in mouse models was determined with reference to other experimental animal studies, which may not represent the most efficacious dosage in the present study. It is also important to note that, in mouse models, BBM showed a better effect than TPT at the early stage (day

4), but less at the late stage (day 8) (**Fig 5C** and **5D**). This difference might be due to individual variation in the rate of drug absorption and metabolism in the animals, which is more complicated than that in cultured cells. By following this critical comment, we have added a few words into the DISCUSION section as follows: "Interestingly, comparative effects of BBM and TPT *in vitro* and *in vivo* appeared to be different. That is, BBM was more effective for inhibiting pseudovirus entry in cultured cells (Fig 4), but was less in mouse models than TPT (Fig 5). This observation might be associated with the differences of these two drugs in optimized dose, and the rate of drug absorption and metabolism in vivo, which awaits further investigation. Taken together, these results strongly suggest a potent inhibitory role of TPT and BBM in SARS-CoV-2 pseudovirus entry." (**Page 17, line 8**)

7. *Recent findings concerning BBM and TPT on COVID-19 should be introduced in more details, and appropriately discussed with the results presented in this study.*

Response: Thank the reviewer for this constructive comment. Following the suggestion, we have added a few sentences to introduce the background and discuss our results and recent findings concerning BBM/TPT on COVID-19 in the DISCUSSION section (**Page 19, line 2**).

8. *The base conservation analysis on SARS-CoV-2 variants should be updated, based on the emergence of novel variants, if possible.*

Response: We thank the reviewer for this important suggestion. As suggested, the conservation analysis on SARS-CoV-2 variants has been updated, incorporating 4 new variants including Omicrion XBB, BQ.1, BA.5 and BA.2.75.2 (**METHODS section, Page 25, line 5**). It revealed that all PQSs are highly conserved and not mutated in all SARS-CoV-2 variants that we examined, suggesting the broad effectiveness of RG4-based therapeutics for SARS-CoV-2 variants.

9. *Some important methods may be described in a little bit more details.*

Response: Thank the reviewer for the suggestion. As suggested, some important methods, especially pseudovirus infection and sequence conservation analysis, have been detailed in the METHODS section (**Page 20, line 20; Page 21, line 12; Page 22, line 17; Page 25, line 5**).

10. *S3 Fig B: "Kiney" should be "Kidney".*

Response: We appreciate the reviewer for careful reviewing. We are so sorry for our carelessness. We have thoroughly checked the manuscript and corrected these typo errors.

Reviewer 2

This is an interesting study. The technical investigations seem fine. The real problem is in the redaction of the article and the conclusions regarding efficacy. This requires completely different experiments and it is highly ambiguous to write that "TPT and BBM block SARS-CoV-2 infection in pseudovirus cell systems and mouse models." SARS-CoV-2 infection is different from pseudovirus infection and a pseudovirus experiment in mice cannot provide a realistic picture of efficacy in vivo.

Therefore,

- either this study positions itself as a fundamental study aimed at highlighting biological mechanisms and characterising them (which it does very well and which is commendable), and part of the article should then be rewritten with this in mind, limiting itself to the results obtained

- or it intends to position itself as a study of the efficacy of molecules with antiviral potential, and it is then necessary to carry out complementary in vitro and in vivo studies with SARS-CoV-2 viruses and an adapted animal model and methodology.

It is important to note that the results presented are not usable to infer drug efficacy, but also that the results presented are not suggestive at this stage of a very high inhibitory potential. The potential use of the drug studied to prevent infection would also deserve a lot of precaution, as the molecules mentioned are not harmless and the benefit/risk balance cannot be assessed from the data provided

Response: We appreciate the reviewer for the favorable and insightful comments.

Major comments

No key new experiment if the paper is limited to the study of pseudoviruses and removes any reference to efficacy. If demonstration of efficacy is the objective:

- antiviral EC50 and EC90 determination in relevant cells (eg TMPRSS2 vero cells + primary explantation bronchial cells) using real virus and different variants

- in vivo experiments in a relevant real virus mouse model or in hamsters

Response: We thank the reviewer very much for these thoughtful and crucial comments. Following these constructive suggestions, we have carefully modified the title, abstract, main text, and Figure legends to emphasize that our manuscript is a fundamental study highlighting biological mechanisms. Basically, we have replaced “SARS-CoV-2 infection” with “SARS-CoV-2 pseudovirus entry”, removed “drug efficacy” concerning our results, limited our conclusions to the results obtained, weakened the clinical potential of our findings, balanced the potential benefit/risk of these two chemicals, and so on. We do hope these modifications considerable and acceptable for the reviewer.

Minor comments

minor edits, eg simulation vs stimulation

Response: We appreciate the reviewer for careful reviewing. This error has been corrected.

Reviewer 3

The approach of developing antiviral therapy, but the experimental design and presented results are not sufficient to support the conclusions.

Response: We appreciate the reviewer for her/his critical and insightful comments.

Major comments

1. *In figure 3, the authors concluded that TPT and BBM inhibited the protein expression levels of some host factors of SARS-CoV-2 by showing western blotting images. To consolidate this conclusion, it is essential to repeat the experiment with sufficient number of replicates, which should enable performing quantification and statistic analysis.*

Response: We appreciate the reviewer for this important comment. As suggested, the results of three repeated experiments were statistically analyzed and presented in the Figures (**Figure below**, red outlines indicating the original image in the manuscript). The replicated data of **Figure 3B-E** are shown in **S2B Fig**.

Fig 3B & S2B Fig

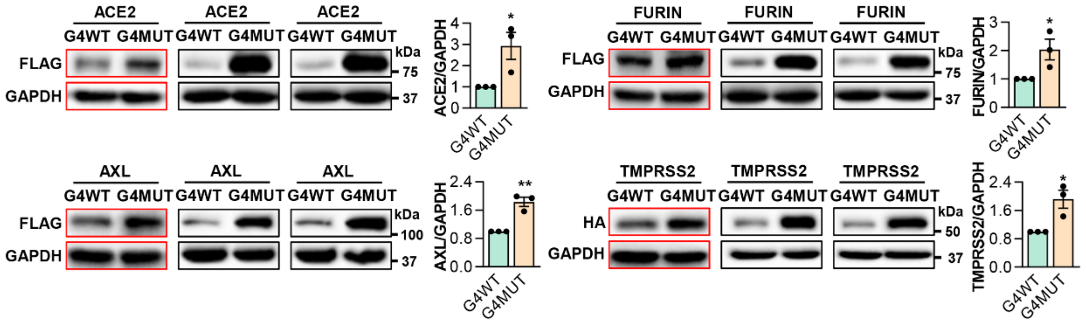

Fig 3C, D & S2B Fig

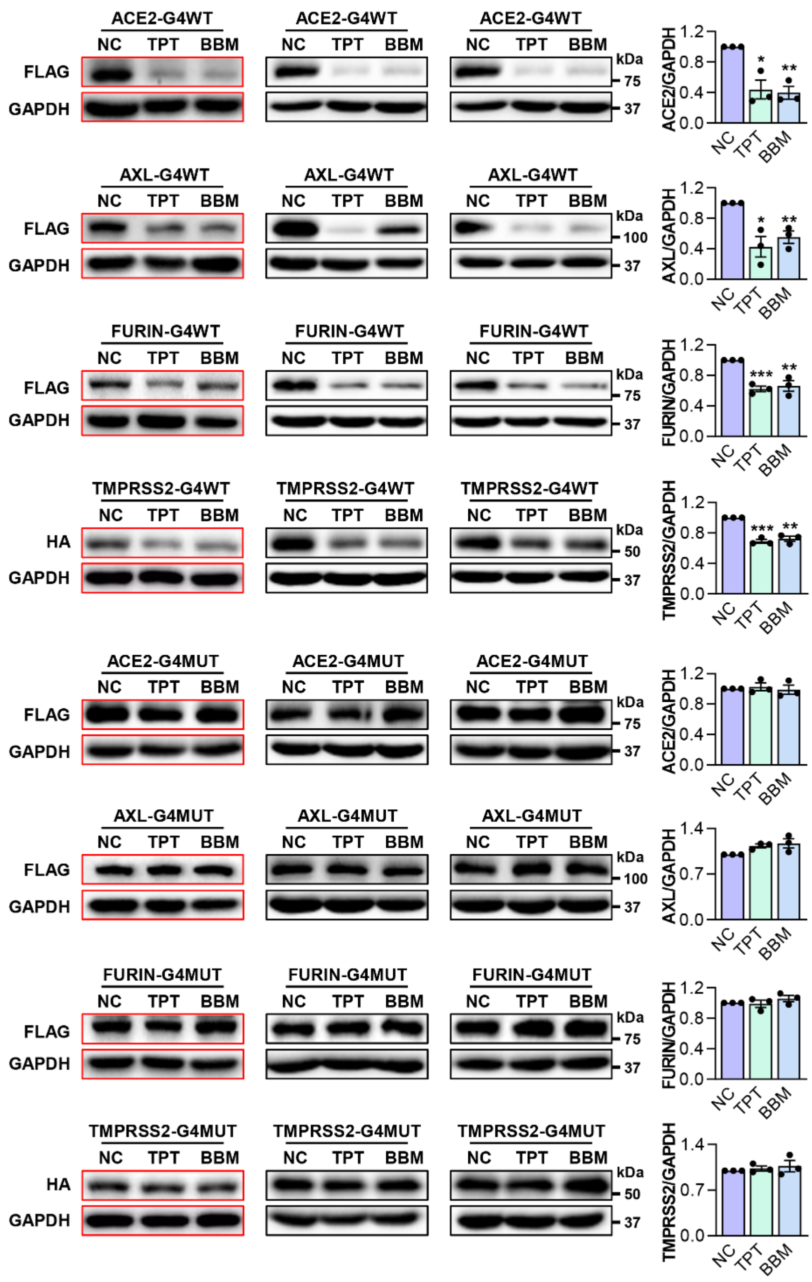

Fig 3E & S2B Fig

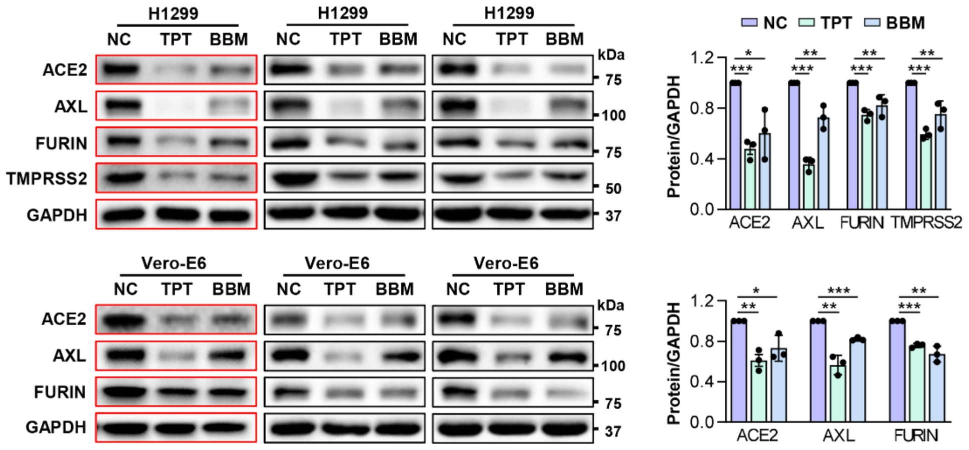

2. *In figure 3, no data actually prove that TPT and BBM indeed inhibit these proteins through targeting RNA G-quadruplex (RG4).*

Response: Thank the reviewer for this thoughtful comment. As shown in **Fig 3C and 3D**, both TPT and BBM diminished the expression of ACE2, AXL, FURIN or TMPRSS2 induced by G4WT plasmids. However, this inhibition was abolished in cells transfected with G4MUT plasmids, in which guanines in G-tracts of target PQSs were substituted with adenines to eliminate RG4 formation with synonymous substitution (**Fig 3A**). Of note, the difference of G4WT and G4MUT plasmid is the former can form RG4 structure, but the latter cannot form RG4 structure due to synonymous substitution. Therefore, these results, together with the high-affinity binding of RG4 structures with TPT and BBM (**Fig 2D-H**), strongly suggest that the repression of ACE2, AXL, FURIN and TMPRSS2 by TPT and BBM depends on RG4 formation. Following this important comment, we have added a sentence in the RESULT section as follows: "<u>which were designed to eliminate the RG4 formation with synonymous substitution</u>" (**Page 10, line 12**), to improve the clarity.

3. *As mentioned in the Introduction, the authors explained that both SARS-CoV-2 and host factors contain RG4. It is evident that many other genes which may or may not relevant to SARS-CoV-2 could also contain RG4. There is no experimental data provided in this study demonstrating which RG4-containing factors (viral or host) actually (partially) mediated the antiviral effects of TPT and BBM. There is only association of TPT and BBM treatment with protein levels of some host factors presented in figure 3.*

Response: We appreciate the reviewer for this crucial and insightful comment. We fully agree with the reviewer that it is of importance to demonstrate the contribution of RG4-containing factors to TPT- and BBM-mediated effects. To address this critical concern, we have performed additional experiments, in which ACE2, AXL, FURIN and TMPRSS2-G4WT plasmids, or their corresponding G4MUT plasmids, were simultaneously transfected into hACE2-

293T cells, followed by SARS-CoV-2 pseudovirus infection. It showed that G4MUT transfection resulted in an increase in pseudovirus entry efficiency, compared with their corresponding G4WT plasmids (**Figure below**). Both TPT and BBM markedly reduced pseudovirus entry in G4WT cells. This inhibition was significantly, but not completely, attenuated in G4MUT cells. These results not only demonstrate that RG4 predominantly mediates the effects of TPT and BBM, but also suggest that certain RG4-independent mechanisms may contribute to the effects of TPT and BBM. Indeed, recent studies indicate that BBM can inhibit SARS-CoV-2 entry by compromising the transient receptor potential mucolipin channels (TRPMLs)-mediated endolysosomal trafficking of ACE2 (***Signal Transduct Target Ther*, 2021, PMID: 33895782**) and blocking S protein-mediated membrane fusion (***PLoS Negl Trop Dis*, 2022, PMID: 35468133**). In this regard, we have revised the DISSCUSION section to discuss the RG4-independent mechanism of TPT and BBM (**Page 18, line 18**). These results are now described in **Fig 4D**.

Fig 4D

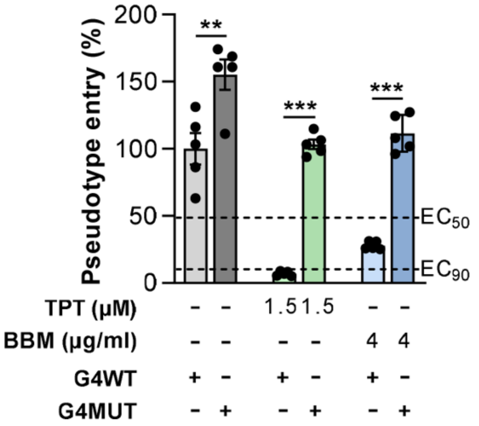

4. *Pseudovirus was used throughout the study. It is essential to be validated using infectious virus strains.*

Response: Thank the reviewer for this important comment, which is also raised by **Reviewer 2**. We totally agree with the Reviewer that using infectious

authentic virus will greatly strengthen the link between BBM/TPT and SARS-CoV-2. However, due to the extraordinary, limited sources and strict control of biological safety, authentic virus assay was extremely difficult for us to conduct at present. However, TPT, in line with another known RG4 stabilizers, has been shown excellent activity to protect against SARS-CoV-2 by using authentic virus in animal models (***Cell*****, 2021, PMID: 33836156;** ***Cell Discov*****, 2022, PMID: 36068208**). These results are consistent with our data, supporting the potential of our strategy for overcoming SARS-CoV-2. Nevertheless, in combination with the suggestions regarding this issue from **Reviewer 2**, we have discussed these limitations in the revised manuscript (**Page 19, line 14**), and modified some sentences to emphasize our current manuscript as a fundamental study. We hope this is considerable and acceptable for the reviewer.

Minor comments

The introduction section, especially the first paragraph can be shortened.

Response: Thank the reviewer for this thoughtful comment. Following the suggestion, we have simplified the introduction section, especially the first paragraph, in the revised manuscript.
