# Supplementary material for: Targeting RNA G-quadruplex with repurposed drugs blocks SARS-CoV-2 entry
Source: PLoS Pathog. 2023 Jan 26;19(1):e1011131. doi: 10.1371/journal.ppat.1011131 (PMC9904497; doi:10.1371/journal.ppat.1011131)
Supplement: Peer review history

# Peer review: Decision Letter 0

**DOI:** 10.1371/journal.ppat.1011131.r001

**Authors:** Ron A. M. Fouchier, Bart L. Haagmans

12 Oct 2022

Dear dr. Xianghui Fu,

Thank you very much for submitting your manuscript "Targeting RNA G-quadruplex with repurposed drugs blocks SARS-CoV-2 infection" for consideration at PLOS Pathogens. As with all papers reviewed by the journal, your manuscript was reviewed by members of the editorial board and by several independent reviewers. In light of the reviews (below this email), we would like to invite the resubmission of a significantly-revised version that takes into account the reviewers' comments.

Although the observations made are of interest, the reviewers raised some important questions especially with regard to the use of pseudotyped viruses to validate their findings. The authors are asked to consider to perform experiments with SARS-CoV-2 viruses in vitro and in vivo.

We cannot make any decision about publication until we have seen the revised manuscript and your response to the reviewers' comments. Your revised manuscript is also likely to be sent to reviewers for further evaluation.

When you are ready to resubmit, please upload the following:

[1] A letter containing a detailed list of your responses to the review comments and a description of the changes you have made in the manuscript. Please note while forming your response, if your article is accepted, you may have the opportunity to make the peer review history publicly available. The record will include editor decision letters (with reviews) and your responses to reviewer comments. If eligible, we will contact you to opt in or out.

[2] Two versions of the revised manuscript: one with either highlights or tracked changes denoting where the text has been changed; the other a clean version (uploaded as the manuscript file).

Important additional instructions are given below your reviewer comments.

Please prepare and submit your revised manuscript within 60 days. If you anticipate any delay, please let us know the expected resubmission date by replying to this email. Please note that revised manuscripts received after the 60-day due date may require evaluation and peer review similar to newly submitted manuscripts.

Thank you again for your submission. We hope that our editorial process has been constructive so far, and we welcome your feedback at any time. Please don't hesitate to contact us if you have any questions or comments.

Sincerely,

Bart L. Haagmans

Guest Editor

PLOS Pathogens

Ron Fouchier

Section Editor

PLOS Pathogens

Kasturi Haldar

Editor-in-Chief

PLOS Pathogens

​orcid.org/0000-0001-5065-158X

Michael Malim

Editor-in-Chief

PLOS Pathogens

orcid.org/0000-0002-7699-2064

***********************

Although the observations made are of interest, the reviewers raised some important questions especially with regard to the use of pseudotyped viruses to validate their findings. The authors are asked to consider to perform experiments with SARS-CoV-2 viruses in vitro and in vivo.

Reviewer's Responses to Questions

**Part I - Summary**

Please use this section to discuss strengths/weaknesses of study, novelty/significance, general execution and scholarship.

Reviewer #1: The manuscript by Tong et al. reports the potential of RG4-targeting drugs on SARS-CoV-2 infection. Initially, the authors screened numerous SARS-CoV-2 host factors by bioinformatic analysis to predict potential RG4s, and then characterized novel RG4s within three host factors, including Ace2, Axl and Furin by a combination of several biochemical and biophysical methods. Subsequently, the authors identified TPT and BBM, two approved clinical drugs, as RG4-stabilizing agents by in silico screening of G4 ligand library and literature searching, followed by microscale thermophoresis validation. Next, the authors performed a series of experiments to verify that RG4s inhibited the expression of ACE2, AXL and FURIN, as well as the previous known RG4-containing TMPRSS2. Importantly, the authors provided strong evidence to demonstrate that both TPT and BBM can effectively repress the expression of ACE2, AXL, FURIN, and TMPRSS2 through the RG4 sequence, and potently reduce the infection of SARS-CoV-2 pseudovirus. Finally, they confirmed the inhibitory effect of TPT and BBM on SARS-CoV infection in mouse models in vivo.

This study not only expands the existence of RG4 in SARS-CoV-2 host factors, but also provides a novel, to my knowledge, RG4-targeting strategy for COVID-19 prevention and therapy. This manuscript is well designed, and the data are solid. Overall, this timely and novel study is of interest and importance to the pathophysiology and treatment of COVID-19, which could offer new insights to beat the ongoing pandemic.

Reviewer #2: This is an interesting study.

The technical investigations seem fine.

The real problem is in the redaction of the article and the conclusions regarding efficacy.

This requires completely different experiments and it is highly ambiguous to write that "TPT and BBM block SARS-CoV-2 infection in pseudovirus cell systems and mouse models." SARS-CoV-2 infection is different from pseudovirus infection and a pseudovirus experiment in mice cannot provide a realistic picture of efficacy in vivo.

Therefore,

- either this study positions itself as a fundamental study aimed at highlighting biological mechanisms and characterising them (which it does very well and which is commendable), and part of the article should then be rewritten with this in mind, limiting itself to the results obtained

- or it intends to position itself as a study of the efficacy of molecules with antiviral potential, and it is then necessary to carry out complementary in vitro and in vivo studies with SARS-CoV-2 viruses and an adapted animal model and methodology.

It is important to note that the results presented are not usable to infer drug efficacy, but also that the results presented are not suggestive at this stage of a very high inhibitory potential.

The potential use of the drug studied to prevent infection would also deserve a lot of precaution, as the molecules mentioned are not harmless and the benefit/risk balance cannot be assessed from the data provided

Reviewer #3: The approach of developing antiviral therapy, but the experimental design and presented results are not sufficient to support the conclusions.

**********

**Part II – Major Issues: Key Experiments Required for Acceptance**

Please use this section to detail the key new experiments or modifications of existing experiments that should be absolutely required to validate study conclusions.

Generally, there should be no more than 3 such required experiments or major modifications for a "Major Revision" recommendation. If more than 3 experiments are necessary to validate the study conclusions, then you are encouraged to recommend "Reject".

Reviewer #1: 1. It has clearly showed that the mutations of RG4 sites can increase the protein levels of exogenous host factors, including ACE2, AXL, FURIN, and TMPRSS2 (Fig. 3A). These data are critical to demonstrate that the formation of RG4 structure can repress the expression of these host factors. However, it remains unclear whether the increase in protein level is due to the difference of transfected plasmid or not. In this regard, the authors should provide the mRNA levels of these host factors after transfection.

2. Both TPT and BBM showed considerable inhibition on ACE2-G4WT expression (Fig. 3B). However, an inhibitory effect was also seen on ACE2-G4MUT expression (Fig. 3C). What is the potential explanation? It is better to perform additional experiments to strengthen this conclusion, if applicable.

Reviewer #2: No key new experiment if the paper is limited to the study of pseudoviruses and removes any reference to efficacy.

If demonstration of efficacy is the objective:

- antiviral EC50 and EC90 determination in relevant cells (eg TMPRSS2 vero cells + primary explantation bronchial cells) using real virus and different variants

- in vivo experiments in a relevant real virus mouse model or in hamsters

Reviewer #3: 1. In figure 3, the authors concluded that TPT and BBM inhibited the protein expression levels of some host factors of SARS-CoV-2 by showing western blotting images. To consolidate this conclusion, it is essential to repeat the experiment with sufficient number of replicates, which should enable performing quantification and statistic analysis.

2. In figure 3, no data actually prove that TPT and BBM indeed inhibit these proteins through targeting RNA G-quadruplex (RG4).

3. As mentioned in the Introduction, the authors explained that both SARS-CoV-2 and host factors contain RG4. It is evident that many other genes which may or may not relevant to SARS-CoV-2 could also contain RG4. There is no experimental data provided in this study demonstrating which RG4-containing factors (viral or host) actually (partially) mediated the antiviral effects of TPT and BBM. There is only association of TPT and BBM treatment with protein levels of some host factors presented in figure 3.

4. Pseudovirus was used throughout the study. It is essential to be validated using infectious virus strains.

**********

**Part III – Minor Issues: Editorial and Data Presentation Modifications**

Please use this section for editorial suggestions as well as relatively minor modifications of existing data that would enhance clarity.

Reviewer #1: 1. It is better to insert S2A-C Fig to Fig. 3. It would be easier for the readers to understand the mutations.

2. The authors should perform the immunohistochemical assay of certain host factors to further confirm the inhibitory effect of TPT and BBM on RG4-containing host factors.

3. Since the in vivo function of TPT and BBM were determined in mouse models, the sequence conservation of the human ACE2, AXL, FURIN RG4s in the corresponding murine mRNAs should be provided. If the sequence conservation is low, the authors should provide experimental data to verify the existence of RG4s within these murine host factors.

4. BBM showed a better effect on SARS-CoV-2 infection than TPT in cultured cells, but seemed to be less than TPT in mouse models. The authors should appropriately discuss this point.

5. Recent findings concerning BBM and TPT on COVID-19 should be introduced in more details, and appropriately discussed with the results presented in this study.

6. The base conservation analysis on SARS-CoV-2 variants should be updated, based on the emergence of novel variants, if possible.

7. Some important methods may be described in a little bit more details.

8. S3 Fig B: “Kiney” should be “Kidney”.

Reviewer #2: minor edits, eg simulation vs stimulation

Reviewer #3: The introduction section, especially the first paragraph can be shortened.

**********

PLOS authors have the option to publish the peer review history of their article (what does this mean?). If published, this will include your full peer review and any attached files.

If you choose “no”, your identity will remain anonymous but your review may still be made public.

**Do you want your identity to be public for this peer review?** For information about this choice, including consent withdrawal, please see our Privacy Policy.

Reviewer #1: No

Reviewer #2: No

Reviewer #3: No

Figure Files:

While revising your submission, please upload your figure files to the Preflight Analysis and Conversion Engine (PACE) digital diagnostic tool, https://pacev2.apexcovantage.com. PACE helps ensure that figures meet PLOS requirements. To use PACE, you must first register as a user. Then, login and navigate to the UPLOAD tab, where you will find detailed instructions on how to use the tool. If you encounter any issues or have any questions when using PACE, please email us at figures@plos.org.

Data Requirements:

Please note that, as a condition of publication, PLOS' data policy requires that you make available all data used to draw the conclusions outlined in your manuscript. Data must be deposited in an appropriate repository, included within the body of the manuscript, or uploaded as supporting information. This includes all numerical values that were used to generate graphs, histograms etc.. For an example see here on PLOS Biology: http://www.plosbiology.org/article/info%3Adoi%2F10.1371%2Fjournal.pbio.1001908#s5.

Reproducibility:

To enhance the reproducibility of your results, we recommend that you deposit your laboratory protocols in protocols.io, where a protocol can be assigned its own identifier (DOI) such that it can be cited independently in the future. Additionally, PLOS ONE offers an option to publish peer-reviewed clinical study protocols. Read more information on sharing protocols at https://plos.org/protocols?utm_medium=editorial-email&utm_source=authorletters&utm_campaign=protocols

# Author comment: Author response to Decision Letter 0

**DOI:** 10.1371/journal.ppat.1011131.r002

16 Dec 2022

Attachment Submitted filename: Response letter to Reviewers.pdf Click here for additional data file.

# Peer review: Decision Letter 1

**DOI:** 10.1371/journal.ppat.1011131.r003

**Authors:** Ron A. M. Fouchier, Bart L. Haagmans

18 Jan 2023

Dear dr Fu,

We are pleased to inform you that your manuscript 'Targeting RNA G-quadruplex with repurposed drugs blocks SARS-CoV-2 entry' has been provisionally accepted for publication in PLOS Pathogens.

Before your manuscript can be formally accepted you will need to complete some formatting changes, which you will receive in a follow up email. A member of our team will be in touch with a set of requests.

Please note that your manuscript will not be scheduled for publication until you have made the required changes, so a swift response is appreciated.

IMPORTANT: The editorial review process is now complete. PLOS will only permit corrections to spelling, formatting or significant scientific errors from this point onwards. Requests for major changes, or any which affect the scientific understanding of your work, will cause delays to the publication date of your manuscript.

Should you, your institution's press office or the journal office choose to press release your paper, you will automatically be opted out of early publication. We ask that you notify us now if you or your institution is planning to press release the article. All press must be co-ordinated with PLOS.

Thank you again for supporting Open Access publishing; we are looking forward to publishing your work in PLOS Pathogens.

Best regards,

Bart L. Haagmans

Guest Editor

PLOS Pathogens

Ron Fouchier

Section Editor

PLOS Pathogens

Kasturi Haldar

Editor-in-Chief

PLOS Pathogens

​orcid.org/0000-0001-5065-158X

Michael Malim

Editor-in-Chief

PLOS Pathogens

orcid.org/0000-0002-7699-2064

***********************************************************

Reviewer Comments (if any, and for reference):

Reviewer's Responses to Questions

**Part I - Summary**

Please use this section to discuss strengths/weaknesses of study, novelty/significance, general execution and scholarship.

Reviewer #1: The authors have satisfactorily addressed all my concerns and revised the manuscript accordingly. Therefore, I recommend publication of this manuscript in Plos Pathogens.

Reviewer #3: The authors have made great efforts to revise and address the comments. The study has been improved.

**********

**Part II – Major Issues: Key Experiments Required for Acceptance**

Please use this section to detail the key new experiments or modifications of existing experiments that should be absolutely required to validate study conclusions.

Generally, there should be no more than 3 such required experiments or major modifications for a "Major Revision" recommendation. If more than 3 experiments are necessary to validate the study conclusions, then you are encouraged to recommend "Reject".

Reviewer #1: (No Response)

Reviewer #3: (No Response)

**********

**Part III – Minor Issues: Editorial and Data Presentation Modifications**

Please use this section for editorial suggestions as well as relatively minor modifications of existing data that would enhance clarity.

Reviewer #1: (No Response)

Reviewer #3: (No Response)

**********

PLOS authors have the option to publish the peer review history of their article (what does this mean?). If published, this will include your full peer review and any attached files.

If you choose “no”, your identity will remain anonymous but your review may still be made public.

**Do you want your identity to be public for this peer review?** For information about this choice, including consent withdrawal, please see our Privacy Policy.

Reviewer #1: No

Reviewer #3: **Yes: **Qiuwei Pan

# Editor report: Acceptance letter

**DOI:** 10.1371/journal.ppat.1011131.r004

**Authors:** Ron A. M. Fouchier, Bart L. Haagmans

23 Jan 2023

Dear Dr. Fu,

We are delighted to inform you that your manuscript, "Targeting RNA G-quadruplex with repurposed drugs blocks SARS-CoV-2 entry," has been formally accepted for publication in PLOS Pathogens.

We have now passed your article onto the PLOS Production Department who will complete the rest of the pre-publication process. All authors will receive a confirmation email upon publication.

The corresponding author will soon be receiving a typeset proof for review, to ensure errors have not been introduced during production. Please review the PDF proof of your manuscript carefully, as this is the last chance to correct any scientific or type-setting errors. Please note that major changes, or those which affect the scientific understanding of the work, will likely cause delays to the publication date of your manuscript. Note: Proofs for Front Matter articles (Pearls, Reviews, Opinions, etc...) are generated on a different schedule and may not be made available as quickly.

Soon after your final files are uploaded, the early version of your manuscript, if you opted to have an early version of your article, will be published online. The date of the early version will be your article's publication date. The final article will be published to the same URL, and all versions of the paper will be accessible to readers.

Thank you again for supporting open-access publishing; we are looking forward to publishing your work in PLOS Pathogens. 

Best regards,

Kasturi Haldar

Editor-in-Chief

PLOS Pathogens

​orcid.org/0000-0001-5065-158X

Michael Malim

Editor-in-Chief

PLOS Pathogens

orcid.org/0000-0002-7699-2064
